# Alteration of microRNA Expression Associated with Chronic Back Pain in Patients with Intervertebral Disc Degeneration: A Scoping Review

**DOI:** 10.3390/ijms27031167

**Published:** 2026-01-23

**Authors:** Azamat V. Ashkhotov, Natalia A. Shnayder, Vera V. Trefilova, Mustafa Al-Zamil, Maxim A. Novitsky, Marina M. Petrova, Natalia P. Garganeeva, Regina F. Nasyrova

**Affiliations:** 1 Institute of Personalized Psychiatry and Neurology, V.M. Bekhterev National Medical Research Centre for Psychiatry and Neurology, 192019 Saint Petersburg, Russia; ashkhotov.v@mail.ru (A.V.A.); vera.v.trefilova@yandex.ru (V.V.T.); regina_nmrcpn@mail.ru (R.F.N.); 2 Neurological Department, United Medical System, 196070 Saint Petersburg, Russia; 3 Shared Core Facilities “Molecular and Cell Technologies”, V.F. Voino-Yasenetsky Krasnoyarsk State Medical University, 660022 Krasnoyarsk, Russia; stk99@yandex.ru; 4 Department of Physiotherapy, Faculty of Continuing Medical Education, Peoples’ Friendship University of Russia, 117198 Moscow, Russia; 5 Neurological Department, Hospital for War Veterans, 193079 Saint Petersburg, Russia; maximnovitsky@gmail.com; 6 Department of General Medical Practice and Outpatient Therapy, Siberian State Medical University, 634050 Tomsk, Russia; garganeeva@gmail.com

**Keywords:** intervertebral disc degeneration, chronic pain syndrome, chronic back pain, systemic inflammation, systemic inflammatory response, local inflammatory response, microRNA, epigenetic biomarker, predictor, protector, prediction, personalized medicine

## Abstract

Chronic back pain (CBP) associated with intervertebral disc degeneration (IVDD) is a leading cause of medical consultations, decreased quality of life, and temporary and permanent disability. The mechanisms of CBP development and persistence in patients with IVDD have been studied for many years, but this issue remains far from resolved. The search for predictive biomarkers that could help identify patients with IVDD at high risk for CBP continues. In recent decades, research has shown increasing interest in identifying epigenetic biomarkers for this disorder. to summarize the results of preclinical and clinical studies on the role of microRNAs (miRs) as epigenetic biomarkers of the development and progression of CBP in patients with IVDD. English-language articles; original experimental (preclinical) studies; original clinical study; assessment of changes in systemic (in the blood) and/or local (in the intervertebral disk (IVD)) levels of miR expression in IVDD, either independently or in comparison with healthy controls; and studies that were completed and the results of which were published. PubMed, Springer, Google Scholar, Scopus, Oxford Press, Cochrane, and e-Library databases. Charting for this scoping review involved developing a data extraction form to summarize extracts and organize data from included studies. This was an iterative process where the charting tables and figures may be refined as the review progresses. 126 studies were analyzed in detail, focusing on their study designs and comparing changes in miR expression in animal models of IVDD and in patients with IVDD compared to healthy controls. During the preparation of this scoping review and upon subsequent detailed review of the original publications, it turned out that the results of one study were not justified by the authors due to identified technological problems (the article was withdrawn by the editorial board of the journal). Therefore, we excluded the results of this study from the subsequent analysis. As a result, this section summarizes the results of 60 preclinical and 65 clinical studies. Some miRs (e.g., miR-21 and miR-132) are associated with the regulation of inflammatory pathways that contribute to increased degradation of IVD extracellular matrix and enhanced nociceptive signaling through various mechanisms, contributing to the progression of CBP. Other miRs (e.g., miR-145 and miR-223) exert protective effects, enhance regenerative potential, and alleviate CBP. Despite the promising results of these studies, there are limitations in the use of miRs as perspective epigenetic biomarkers of CBP in patients with IVDD because the pattern of potentially predictive and protective miRs in relation to the mechanisms of CBP formation and progression in IVDD has not yet been sufficiently studied. The results of some preclinical and clinical studies are contradictory. Further research is needed to clarify the role of miR signatures in animal models and clinical trials on IVDD-specific CBP.

## 1. Introduction

Intervertebral disc degeneration (IVDD) is a multifactorial, chronic, recurring process that plays a significant role in the development of acute and chronic back pain (CBP). This is due to the additive effect of environmental factors (metabolic patterns, excess or insufficient body weight, physical activity, climatic and geographical conditions, ethnic factors, psychosocial factors, etc.) and genetic predisposition [1]. There is no unified hypothesis regarding the mechanisms underlying the development of acute pain or CBP in IVDD, as the same structural instability of the spine and herniated intervertebral discs (IVD) can be identified in asymptomatic individuals. Therefore, degenerative changes in the structure and stability of the spine may not be directly related to discogenic back pain [2]. In most modern scientific studies, the formation and maintenance of CBP in IVDD is attributed to a systemic and local inflammatory response associated with the production of proinflammatory mediators [2]. On the one hand, overproduction of proinflammatory mediators directly or indirectly disrupts the natural processes of regeneration and repair of IVD structures and supports the processes of neo-angiogenesis and neurogenesis with the ingrowth of new vascular–neuronal complexes for the process of IVD adaptation to conditions of hypoxia and inflammation. On the other hand, overproduction of proinflammatory mediators provokes the development of CBP [3].

Epigenetic processes and their influence on the expression of a particular trait encoded by the corresponding gene are of increasing interest to researchers. The molecular basis of epigenetics does not affect the primary structure of genes contained in deoxyribonucleic acid (DNA), but modifies the activity of certain genes. This explains why, in IVDD, only genes necessary for preventing disease progression and its unfavorable course are expressed [2]. Within the framework of epigenetics, various biomarkers are being studied, among which short single-stranded ribonucleic acids (microRNAs) are of the greatest scientific interest. Patients with IVDD are characterized by interindividual variability in the expression level of microRNAs (miRs), which can modulate the activity of genes associated with the progression or improvement of CBP [4]. Also, updating knowledge about the role of miRs is important to improve our understanding of the epigenetic mechanisms that influence the initiation and maintenance of local and systemic inflammatory responses associated with the progression of degenerative processes and herniation in patients with IVDD.

It is known that microRNAs are a class of non-coding single-stranded RNAs of 19–24 nucleotides that can post-transcriptionally modulate a significant part of the genome [4,5] by binding to the 3′-untranslated region (UNR), and sometimes to the 5′-untranslated region (UNR). This allows one specific miR to inhibit the translation of several genes, so miRs can modulate numerous pathophysiological processes in IVDD (acute back pain, CBP and other related disorders) [4].

Based on current knowledge and understanding of the role of the chronic inflammatory response in IVDD, standard and novel drug therapies [6,7,8], physical therapy, and, in extreme cases, invasive surgical procedures such as spinal fusion or arthroplasty [9], are used. Due to the major limitations of these treatments for CBP in patients with IVDD, including modest success rates, invasiveness, and high cost, there is a high demand for new, targeted therapies to correct the chronic inflammatory response, counteract degenerative processes in IVD, and reduce back pain [8]. The most promising of these include cell-based therapies, endogenous repair strategies through the activation of IVD reparative cells, and treatments based on biological factors, including the use of miRs [8,10]. For example, some miRs are selectively excreted through lipoproteins or microvesicles, acting as a mode of intercellular communication. This concept is crucial regarding the potential of microRNAs not only as specific biomarkers of IVDD but also as potential treatments for many processes related to cellular homeostasis in IVD and surrounding tissues [2].

Altered protein expression is one of the key characteristics of the development of long-term hyperexcitability of peripheral nociceptive and central neurons, which may contribute to the development or maintenance of CBP in patients with IVDD [11]. This process is inherently subject to potential regulation by miRs, both centrally and peripherally. Thus, in the periphery, proinflammatory mediators (prostaglandin E2 (PGE2) and interleukin 1 beta (IL1β)) promote the activation of tumor necrosis factor alpha (TNFα) in immune cells, growth factors (granulocyte–macrophage colony-stimulating factor—GM-CSF, nerve growth factor—NGF), and neuropeptides (calcitonin gene-related peptide—CGRP, substance P, and histamine from local nociceptors), which are involved in the development or maintenance of hyperalgesia in IVDD [12]. These components of local and systemic inflammation stimulate the activity of gene pathways, including MAP/microtubule affinity-regulating kinase 3 and Janus kinase-signal transducer and activator of transcription Janus kinase-STAT signaling, and recruit multiple downstream enzymes, such as phospholipase C and phosphoinositide 3-kinase. This induces de novo phosphorylation and transcription of nociceptive molecules (members of the subfamily V cationic channels transient receptor potential 1 and Nav1.8 (a subtype of sodium ion channels), which leads to hyperexcitability of nociceptive Aδ- and C-fibers (nociceptive-specific) and maintenance of CBP in patients with IVDD [12].

Central sensitization is mediated by synaptic plasticity, glial cell activation, decreased pain thresholds, and decreased endogenous modulation [13]. Excitatory synaptic communication between first-order neurons and spinal cord neurons is largely facilitated by the neurotransmitter glutamate and modulated by factors such as calcitonin and brain-derived neurotrophic factor (BDNF) [14]. Changes in spinal motion segment function include long-term synaptic strengthening and increased neuronal and glial hyperactivity or hyperreactivity in the spinal dorsal horn, leading to an overall increase in pain sensitivity in patients with IVDD [11,15].

Genetic, epigenetic, and transcriptional changes are expressed at the protein level, and miRs are involved in the post-transcriptional regulation of proteins critical for pain processing pathways from degenerating IVDs and its perception in the brain [2]. Dysregulation of miRs targeting key regulators of pain processing (gamma-aminobutyric acid-α1 (GABA-α1) [16], cyclooxygenase 2 type (COX2) [17], vanilloid receptor 1 [5], and voltage-gated Na^+^ and Ca^2+^ ion channels [18]) has been observed in various surrogate pain models. Similarly, miR-23b has been shown to regulate M-opioid receptor type 1 expression [19], and opioid tolerance is potentially a consequence of miR-23b upregulation [20].

The rationale for this scoping review was the increasing evidence that microRNAs are involved in the pathological processes of IVDD (including pain and inflammation), making them promising candidates for epigenetic diagnostic biomarkers and therapeutic targets. While many miRs have been implicated in IVDD through their role in apoptosis, extracellular matrix (ECM) remodeling, and inflammation, further testing is needed to identify specific miRs for early diagnosis, stratify patients with IVDD (high and medium risk groups), develop new treatments (disease-modulated miRs as drugs of new generation), and confirm their causal role in developing CBP in patients with IVDD.

Given the heterogeneity of the designs of previously conducted preclinical and clinical studies on the topic of our interest, we chose a scoping review. Our choice is due to the fact that this type of review is aimed at broadly displaying and summarizing existing research in a particular field in order to identify key concepts, types of research, and knowledge gaps. This scoping review is our first step before a narrow systematic review. In addition, a scoping review may help to understand the overall picture of current research of miRs as epigenetic biomarkers of CBP in patients with IVDD. In contrast to a narrowly focused systematic review, in our scoping review, we did not aim to answer a specific question, but rather to determine the scale and nature of the available data, to be a “telescope” covering a wide area of epigenetic research of IVDD, rather than a “microscope”.

The main aim of this scoping review is to summarize the results of preclinical and clinical studies on the role of miRs as perspective epigenetic biomarkers of the development and progression of CBP in patients with IVDD.

## 2. Materials and Methods

In this study, we aim to present the current state of knowledge regarding the utility and potential limitations of using miRs as epigenetic biomarkers in diagnostic protocols for IDD. We also attempt to answer a scientifically and clinically important question: “Could the miR signature replace (as an effective alternative with a better prognosis) or enhance (as an additional technique) diagnostic laboratory tests in the future?”.

A search was conducted in the PubMed, Springer, Google Scholar, Scopus, Oxford Press, Cochrane, and e-Library databases. The search was conducted using the following keywords and phrases: microRNA, intervertebral disc degeneration, chronic pain syndrome, low back pain, epigenetics, systemic inflammatory response syndrome, diagnosis, and treatment. Publications from 2015 to 2025 were analyzed, including original clinical studies involving patients with CBP and signs of systemic inflammatory response syndrome. In addition, publications of clinical and historical interest (published no earlier than 2005) were included.

We excluded articles that were case reports or reviews, as well as studies that were still ongoing.

So, the studies analyzed in this scoping review had to meet the following inclusion criteria:

- English-language articles,
- Original experimental (preclinical) studies,
- Original clinical studies,
- Assessments of changes in systemic (in blood) and/or local (in IVD and other tissue) levels of miR expression in IVDD, either independently or in comparison with healthy controls,
- Studies that were completed and the results of which were published.

A total of 365 publications were analyzed. After excluding duplicate publications, 60 preclinical and 66 clinical studies meeting the objectives and search criteria of PRISMA-ScR (Preferred Reporting Items for Systematic reviews and Meta-Analyses extension for Scoping Reviews) [21] were included in this scoping review (for the PRISMA-ScR list, please refer to the Supplementary Materials). Our review has not been assigned a registration number in PRISMA 2020, as registration for the scoping review is not mandatory.

Circulating miR studies were conducted using blood samples, including serum, plasma extracellular vesicles (exosomes), mononuclear cells, and tissue samples of degenerated IVDs obtained after surgical treatment of hernias (discectomy). miRs were identified as epigenetic biomarkers of CBP development (pathogenesis), CBP severity, and new therapeutic strategies for CBP in patients with IVDD.

The analysis focused primarily on original experimental preclinical and clinical studies assessing changes in the expression level of miRs in IVD and other tissue and peripheral blood (local and circulating miRs, respectively).

Before charting, we determined the specific information needed to answer the review’s objective. This included study details, samples characteristics, intervention types, and reported outcomes. When selecting preclinical studies, we evaluated IVDD-specific models of CBP and non-IVDD-specific models of CBP, and local (tissue) and circulating miRs. When selecting clinical studies, we evaluated the sample size, age of participants, and study design, tissue, and circulating miRs. Charting tables for this scoping review involved creating a standardized data extraction form to summarize, collect, and organize information from included studies. Also, we decided on the appropriate level of detail for each data point. Some information, like author and year, were standard, while other fields required more in-depth extraction. We considered using data visualizations like concept figures to present the findings of this review in a clear and logical manner. As we charted, we identified additional useful data points that could be added to the figures, continually updating it as the review progressed.

This scoping review was based on the study and synthesis of available publications relevant to the topic and aim of our study, identifying key concepts, theories, and gaps in miR research as epigenetic biomarkers of pain and inflammation in patients with intervertebral disc degeneration. Unlike other types of reviews (in particular, a scoping review, a systematic review, or meta-analysis), our scoping review focused on reviewing and mapping the entire data set, rather than answering a specific research question, that is, it was used as the first step to determine the directions of future research.

We did not use statistical data processing and plotting methods, since the analyzed preclinical and clinical studies were characterized by great variability in the design, type and sample sizes.

**Figure 1:**
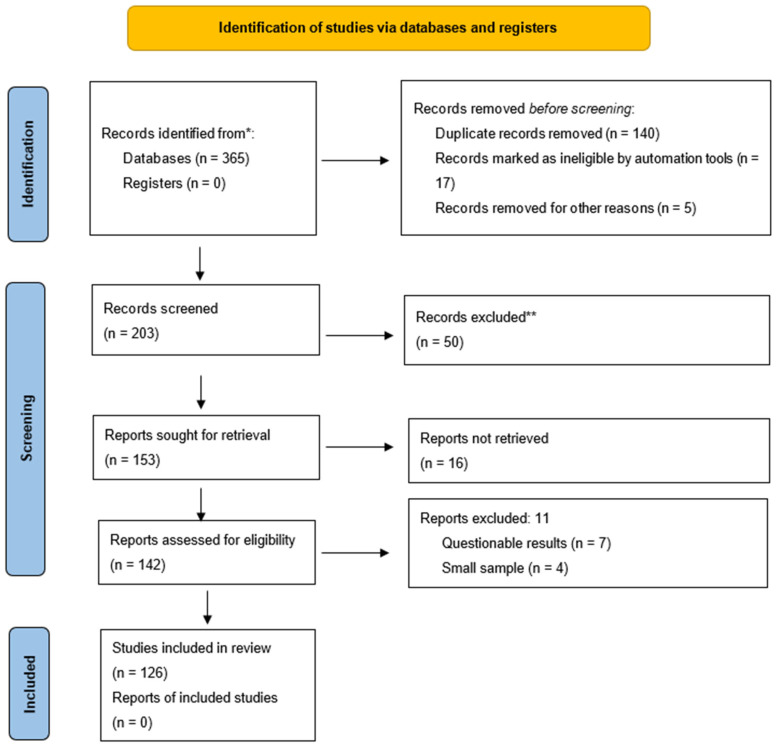
Identifications of analyzed studies by databases (PRISMA-ScR) [21]. Note: * The total number of records identified from all databases and registers; ** The total number of records were excluded by a human.

The number of verified evidence sources assessed for compliance and included in the review, with reasons for exclusion at each stage, is presented in this scoping review using a flow chart (Figure 1).

## 3. Results

Based on the inclusion and exclusion criteria, we analyzed 126 studies. The studies were analyzed in detail, focusing on their study designs and comparing changes in miR expression in animal models of IVDD and in patients with IVDD compared to healthy controls. During the preparation of this scoping review and upon subsequent detailed review of the original publications, it turned out that the results of one study were not justified by the authors due to identified technological problems (the article was withdrawn by the editorial board of the journal). Therefore, we excluded the results of this study from the subsequent analysis. As a result, this section summarizes the results of 60 preclinical and 65 clinical studies.

### 3.1. Preclinical Studies

Most preclinical studies have been conducted using rodent models of back pain caused by compression, injury, or ligation of lumbar spinal roots. Some studies have been conducted using models of oxidative-inflammatory response, knee osteoarthritis, and cultured peripheral blood cell lines and IVD nucleus pulposus (NP) cells [22,23]. Polymerase chain reaction, real-time polymerase chain reaction, or microRNA microarray were used to study miR expression and downregulation.

According to the studies presented in Table 1, most previously studied miRs are associated, through various mechanisms, with the activation and maintenance of a systemic inflammatory response in IVDD. The most studied epigenetic biomarkers are miR-1, miR-21, miR-23C, miR-23a-3p, miR-26a-5p, miR-30c-5p, miR-34c, miR-125b-5p, miR-132, miR-142-3p, miR-148a, miR-155, miR-183, miR-218, miR-221, miR-341, miR-431, miR-431-5p, miR-483-3p, and miR-511-3p [24,25,26,27,28,29,30,31,32,33,34,35,36,37,38,39,40,41,42]. Other biomarkers have protective properties against systemic inflammatory response, including miR-15A-5p, miR-16, miR-31-5p, miR-34c, miR-124, miR-125b, miR-132, miR-138, miR-143-5p, miR-145, miR-146a, miR-146a-5p, miR-149, miR-183, miR-185, miR-186-5p, miR-190a-5p, miR-200b, miR-221, miR-223, miR-378, miR-381, miR-429, miR-590-3p, and miR-874-3p [22,23,29,36,43,44,45,46,47,48,49,50,51,52,53,54,55,56,57,58,59,60,61,62].

It is known that miRs (largely due to the phenomenon of peripheral and central sensitization) modulate the increase or decrease in CBP depending on the proinflammatory or anti-inflammatory effects [11]. At the same time, the processes of facilitating the regulation of genes responsible for the formation of a systemic inflammatory response deserve special attention, thanks to the following biomarkers: miR-15b, miR-17-92, miR-23b, miR-30b, miR-30b-5p, miR-96, miR-103, miR-122-5p, miR-132, miR-132-3p, miR-181a, miR-182, miR-200b, miR-429, miR-431-5p, miR-483-3p, miR-500, miR-let-7a, and miR-let-7b [16,39,42,44,45,63,64,65,66,67,68,69,70,71,72,73,74,75,76]. Furthermore, hypo-expression of miR-200b and miR-429 is associated with elevated levels of methyltransferase 3A, which is involved in the generation of dysfunction of the mesolimbic motivation/evaluation circuitry, linking prolonged nociceptive stimuli with comorbidities such as anxiety and sleep disorders [73]. In turn, anxiety–depressive disorders can maintain and exacerbate CBP in patients with IVDD [77].

The mechanisms of action of miRs in the epigenetic modification of the IVDD process (according to preclinical studies) are presented in Table 1.

**Table 1:** Preclinical studies of the role of microRNA in development of chronic back pain in patients with intervertebral disc degeneration.

MicroRNA	Design of the Study	Tissue (Simple)	General Characteristics of the Sample	Effect of the microRNA	References
A. IVDD-specific model of chronic pain					
Local microRNA					
miR-15A-5p	Experimental (mice)	IVD tissue	Model of traumatic needle injury	Overexpression of miR-15A-5p facilitates NP cell proliferation and promotes SOX9 expression to suppress inflammatory response and apoptosis via NF-κB signaling pathway.	[41]
miR-16	Experimental (Sprague– Dawley rats)	NP tissue	Model of inflammatory injury	Overexpression of miR-16 modulates the inflammatory response in NP cells stimulated with LPS; miR-16 activates the expression of ECM genes (aggrecan and collagen II), but inhibits the genes of MMP3, MMP13, disintegrin, and ADAMATS4 and ADAMATS5 through the NF-κB and MAPK signaling pathway by targeting their upstream TGF-β-activated kinase 1 and MAP3K7-binding protein 3.	[59]
miR-21	Experimental (Sprague– Dawley rats)	IVD tissue	Surgical model of IVD tissue injury	Overexpression of miR-21 targets PTEN, implicating it in abnormal NP cell proliferation through suppression of the AKT pathway; miR-21 inhibitors can reduce the expression of inducible HIF-1α and VEGF, inhibiting NP cell apoptosis.	[38]
miR-31-5p	Experimental (mice)	IVD tissue	Model of traumatic injury	Overexpression of miR-31-5p induces NP cell proliferation, inhibits apoptosis, and promotes ECM formation by regulating the SDF-1/CXCR7 pathway.	[60]
	Experimental (Sprague– Dawley rats)	IVD tissue	Model of inflammatory injury	Overexpression of miR-31-5p inhibits apoptosis in endplate chondrocytes via regulation of ATF6.	[61]
miR-132	Experimental (rats)	IVD tissue	Rat tail needle injury model	Overexpression of miR-132 induces ECM degradation by directly targeting GDF5 and leading to increased expression of MMP13 and ADAMTS4 via the MAPK/ERK pathway.	[31]
miR-143-5p	Experimental (rats)	IVD tissue	Model of puncture needle injury	Overexpression of miR-143-5p induces apoptosis and proliferation of IVD cells via the AMPK signaling pathway.	[47]
miR-145	Experimental (rats)	NP tissue	Model of oxidative stress in cultured cell lines	Overexpression of miR-145 targeting ADAMTS 17 inhibits NP IVD cell apoptosis in vitro, both in the presence and absence of oxidative stress.	[22]
miR-148a	Experimental (Sprague– Dawley rats)	IVD tissue	Model of injection injury	Overexpression of miR-148a induces increased Hotair activity, which in turn increases PTEN expression, leading to the progression of IVDD.	[41]
miR-149	Experimental (rats)	NP cell	Inflammation model using liposomes	Overexpression of miR-149 induces hyperproduction of MMP-3, ADAMTS4, and other inflammatory cytokines via MyD88.	[50]
miR-155	Experimental (rats)	NP cells	Model of inflammatory injury by treatment with TNF-α or IL-1β	Overexpression of miR-155 inhibits ECM degradation via p65/NF-κB signaling.	[33]
miR-181a	Experimental (mice)	NP tissue	Model of NP tissue injury by needle puncture	Overexpression of miR-181a inhibits the inflammatory response by inactivating the ERK pathway.	[78]
miR-185	Experimental (Sprague– Dawley rats)	NP tissue	Model of IVDD (unspecified)	Overexpression of miR-185 targets galectin 3, a β-galactosidase-binding protein involved in apoptosis and the Wnt/β-catenin pathway.	[51]
miR-431-5p	Experimental (Sprague– Dawley rats)	IVD tissue	Model of traumatic needle injury	Overexpression of miR-431-5p inhibits Circarl15 production in IDD tissues (Circarl15 expression was positively correlated with the DISC1 scaffold protein; Circarl15 suppresses NP cell apoptosis but promotes NP cell proliferation through regulation of the miR-431-5p/disc1 signaling axis).	[42]
miR-874-3p	Experimental (rats)	NP tissue	Model of needle puncture trauma	Overexpression of miR-874-3p targets ATF3; inhibits NP cell apoptosis by reducing caspase-3 degradation; inhibits ECM degradation by reducing the catabolic factors MMP2 and MMP3.	[62]
Circulating microRNA					
miR-23C	Experimental (rats)	Blood	Model of IVDD (unspecified)	Overexpression of miR-23c and CTNNB1 is associated with the development and progression of IVDD.	[39]
miR-26a-5p	Experimental (mice)	Blood (serum)	Model of traumatic injury IVDD	Overexpression of miR-26A induces the development of IVDD, acting as one of the possible biomarkers of the disease.	[40]
miR-483-3p	Experimental (rats)	Blood	Model of IVDD (unspecified)	Overexpression of miR-483-3p and GSK3B induces the development and progression of IVDD.	[39]
B. Non-IVDD-specific model of chronic pain					
Local microRNA					
miR-1	Experimental (rats)	Dorsal spinal root ganglia	Model of unilateral chronic sciatic nerve injury and compression	Overexpression of miR-1 increases Cx43/BDNF expression, which in turn leads to the formation of CBP.	[24]
miR-17-92	Experimental (rats)	L5 posterior spinal root ganglia	Injury model of L5 root ligation; model of mechanical allodynia by local injection of adeno-associated virus vector	Overexpression of miR-17-92 inhibits the expression of potassium channels and reduces extracellular potassium currents (especially A type), alleviating mechanical allodynia.	[64]
miR-21	Experimental (mice)	Posterior spinal root ganglia	Partial ligation model of the sciatic nerve	Overexpression of miR-21 induces IL6 synthesis and the development of neuropathic pain syndrome.	[25]
		Posterior spinal root ganglia	Model of spinal nerve ligation	Overexpression of miR-21 acts on TLR8 in lysosomes as an endogenous ligand, inducing activation of ECM degradation and production of inflammatory mediators, promoting CBP formation.	[79]
miR-23a-3p	Experimental (mice)	Mouse spinal cord neurons. Sciatic nerve	Partial ligation/injury model of the sciatic nerve	Overexpression of miR-23a-3p induces neuropathic pain by directly targeting CXCR4 via the TXNIP/NLRP3 inflammasome axis.	[26]
miR-23b	Experimental (mice)	Spinal cord (neurons)	Model of chronic spinal nerve injury	Overexpression of miR-23b targets NOX4, which reduces the production of the inhibitory neurotransmitter GABA and promotes the formation of mechanical and thermal hyperalgesia.	[65]
miR-26a-5p	Experimental (rats)	Spinal cord (neurons)	Model of chronic spinal nerve injury	Overexpression of miR-26a-5p acts as a negative regulator of neuropathic pain development via targeting MAPK6.	[27]
miR-30b	Experimental (rats)	Posterior spinal root ganglia	Model of L5 root ligation	Overexpression of miR-30b reduces neuropathic pain by inhibiting the expression of voltage-gated sodium channels Nav1.3.	[66]
miR-30b-5p	Experimental (rats)	Posterior spinal root ganglia	Model of peripheral neuropathic pain (by intraperitoneal administration of oxaliplatin)	Overexpression of miR-30b-5p induces NP degeneration via suppression of Nav1 sodium ion channels.	[67]
miR-30c-5p	Experimental (anti-allodyn phenotype of mice)	Spinal cord (neurons). Dorsal spinal root ganglia (neurons). Cerebrospinal fluid.	Chronic sciatic nerve injury model	Overexpression of miR-30c-5p induces the development of neuropathic pain via BAMBI (TGFβ pseudo-receptor).	[28]
miR-34c	Experimental (mice)	Spinal cord (neurons)	Model of sciatic nerve constriction/compression	Overexpression of miR-34c inhibits the production of NLRP3, caspase-1, IL1β, and IL18, and reduces neuropathic pain.	[29]
miR-96	Experimental (rats)	Posterior spinal root ganglia (neurons)	Model of chronic sciatic nerve compression	Hypo-expression of miR-96 activates sodium channels Nav1.3, which are involved in the formation of CBP.	[68]
miR-103	Experimental (rats)	Posterior spinal root ganglia (neurons)	Model of chronic injury	Overexpression of miR-103 reduces neuropathic pain through a bidirectional and integrated regulatory role in Cav1.2-LTC calcium channels.	[69]
miR-122-5p	Experimental (rats)	Spinal cord (neurons)	Model of chronic sciatic nerve injury	Overexpression of miR-122-5p inhibits the mechanism of neuropathic pain development via PDK4.	[70]
miR-124	Experimental (mice)	Spinal cord (neurons)	Model of peripheral inflammatory hyperalgesia	Overexpression of miR-124 modulates microglial activity by affecting MeCP2 and reducing central pain sensitization.	[43]
		Umbilical cord (microglia)	IL-1β-induced inflammatory hyperalgesia model in mice with chronic carrageenan-induced hyperalgesia	Overexpression of miR-124 shifts the “pro-inflammatory M1/anti-inflammatory M2” balance toward the anti-inflammatory M2 phenotype and reduces mechanical hyperalgesia and pain behavior.	[80]
miR-125b-5p	Experimental (rats)	Masseter muscle. Trigeminal ganglion	Model of ligation of spinal roots L4 and L5	Overexpression of miR-125b-5p induces the development of neuropathic pain by activating *TNF* and *BDNF* genes.	[29]
miR-125b	Experimental (rats)	Brain (hippocampal neurons)	Model of chronic constriction injury of spinal roots	Hypo-expression of miR-125b induces CBP development in peripheral nerve injury through NR2A regulation by altering BDNF expression.	[44]
miR-132	Experimental (rats)	Brain (hippocampal neurons)	Model of chronic constriction injury of spinal roots	Hypo-expression of miR-132 induces CBP development in peripheral nerve injury via regulation of BDNF and NR2A expression (by altering BDNF expression).	[44]
	Experimental (MeCP2 transgenic mice)	Spinal cord (neurons)	Model of peripheral nerve injury	Overexpression of miR-132 via the P-Creb/miR-132 signaling cascade induces the development of MeCP2-mediated pain.	[45]
miR-132-3p	Experimental (rats)	Posterior spinal root ganglia (neurons). Spinal cord (neurons)	Model of spinal root and nerve damage	Overexpression of miR-132-3p induces the development of neuropathic pain and CBP via upregulation of AMPA receptor subunits GluA1 and GluA2 in the spinal cord.	[71]
miR-138	Experimental (rats)	Spinal cord (neurons)	Sciatic nerve ligation	Overexpression of miR-138 inhibits neuropathic pain by suppressing TLR4 and MIP-1α/C signaling pathway CCR1.	[46]
miR-142-3p	Experimental (rats)	Sciatic nerve. Spinal cord (neurons)	Model of sciatic nerve injury	Hypo-expression of miR-142-3p induces an increase in the expression of AC9 and cAMP, which leads to a decrease in the level of proinflammatory mediators and a decrease in neuropathic pain (due to an increase in the expression of proteins associated with the CAMP/AMPK pathway, which regulates energy and redox homeostasis).	[32]
miR-146a	Experimental (rats)	Posterior spinal root ganglia (neurons)	Model of knee osteoarthritis	Overexpression of miR-146a in astrocytes and microglia inhibits the formation of proinflammatory transcripts (TNFα, COX2, iNOS, and IL6) that influence the formation of CBP.	[81]
	Experimental (rats)	Posterior spinal root ganglia (neurons). Spinal cord (neurons of the dorsal horn). Knee joint (medial meniscus tissue)	Surgical model of osteoarthritis (medial meniscus transection)	Hypo-expression of miR-146a activates proinflammatory pain mediators and induces mechanisms of subsequent loss of glial function.	[48]
miR-146a-5p	Experimental (rats)	Posterior spinal root ganglia (neurons). Spinal cord (neurons of the posterior horn)	Model of chronic sciatic nerve injury	Overexpression of miR-146a-5p regulates neuropathic pain by inhibiting IRAK1 and TRAF6 in TIR; inhibits JNK/CCL2.	[49]
miR-155	Experimental (rats)	Brain (neurons of prefrontal cortex)	Model of peripheral inflammatory hyperalgesia induced by carrageenan injection	Overexpression of miR-155 inhibits CEBPB production but induces GCSF production, which is accompanied by increased immunolabeling of antibodies to myeloperoxidase, which increases inflammation and activates the prefrontal cortex, promoting the formation of CBP.	[82]
miR-181a	Experimental (rats)	Posterior spinal root ganglia (neurons)	Model of peripheral inflammatory hyperalgesia using zymosan	Overexpression of miR-181a inhibits transcriptional regulation of the GABAergic system in CBP (post-transcriptional suppression of the developing spinal GABAergic system).	[16]
miR-181a-3p	Experimental (rats)	Sciatic nerve	Model of chronic bilateral sciatic nerve compression	Hypo-expression of miR-181a-3p induces the development of neuropathic pain.	[37]
miR-182	Experimental (rats)	Posterior spinal root ganglia (neurons)	Model of chronic neuropathic pain (L5 spinal root ligation)	Hypo-expression of miR-182 induces the distribution of SG and TIA1.	[72]
miR-183	Experimental (rats)	Lumbar spinal roots ganglia (neurons). Spinal cord (neurons of the posterior horn). Knee joint (medial meniscus tissue)	Surgical model of osteoarthritis (medial meniscus transection)	Hypo-expression of miR-183 induces proinflammatory pain mediators and enhances subsequent loss of glial function.	[48]
		Spinal cord (neurons of the dorsal horn)	Model of chronic injury/compression	Hypo-expression of miR-183 inhibits mechanisms of neuropathic pain development through the blockade of serine–threonine protein kinase receptors mTOR and VEGF.	[23]
		Spinal cord (tissue). Spinal cord (neurons)	Model of chronic sciatic nerve injury	Overexpression of miR-183 targets MAP3K4, which inhibits proinflammatory cytokines (IL-6 and IL-1β) and COX2.	[34]
miR-186-5p	Experimental (mice)	Spinal cord (tissue). Spinal cord (astrocytes)	Model of spinal root ligation	Overexpression of miR-186-5p inhibits CXCL13 expression, alleviating neuropathic pain.	[52]
miR -190a-5p	Experimental (mice BALB/c)	Spinal cord, lumbar level (tissue of dorsal horn)	Model of diabetic neuropathic pain	Overexpression of miR-190a-5p and inhibition of SLC17A6 can significantly attenuate neuropathic pain and reduce the production of proinflammatory cytokines IL1β and IL6.	[53]
miR-200b	Experimental (mice)	Sciatic nerve	Model of unilateral partial ligation of the sciatic nerve	Hypo-expression of miR-200b induces DNMT3a production, which is involved in generating dysfunction of the mesolimbic motivation/evaluation circuitry that links prolonged nociceptive stimuli with comorbidities in CBP (anxiety and sleep disorders).	[73]
	Experimental (rats)	Spinal cord (tissue). Isolated microglial cells	Model of chronic damage and compression of spinal roots	Overexpression of miR-200b inhibits proinflammatory cytokines (IL6, IL1β, and TNFα) by targeting ZEB1.	[54]
miR-203	Experimental (rats)	Sciatic nerve	Model of chronic bilateral compression of the sciatic nerves	Hypo-expression of miR-203 induces the development of neuropathic pain.	[37]
miR-218	Experimental (rats)	Spinal cord (tissue). Isolated microglial cells	Model of chronic spinal root injury	Hypo-expression of miR-218 inhibits mechanical allodynia, thermal hyperalgesia, and proinflammatory cytokine release via SOCS3, regulates SOCS3 mRNA and protein expression, and inactivates the transducer JNK and STAT3.	[35]
miR-221	Experimental (rats)	Spinal cord (tissue). Isolated microglial cells	Model of chronic spinal root injury	Hypo-expression of miR-221 inhibits mechanical allodynia and thermal hyperalgesia, and inhibits the expression of proinflammatory cytokines (TNFα, IL1β, and IL6) through SOCS1, as well as through suppression of NF-κB activation and the p38-MAPK signaling pathway.	[83]
miR-223	Experimental (mice)	Spinal cord (neurons)	Model of chronic spinal root injury	Overexpression of miR-233 inhibits the mechanisms of neuropathic pain development through inhibition of NLRP3 expression; reduces the levels of NLRP3, ASC, caspase-1, IL1β, and IL18; increases the proportion of M2 macrophages and decreases the proportion of M1 macrophages.	[55]
	Experimental (rats)	Spinal cord (neurons)	Model of chronic injury/compression/of spinal roots; model of inflammatory-oxidative reaction of NP cells	Overexpression of miR-223 inhibits the expression of NLRP3, associated with apoptosis, as well as caspase-1, IL1β, and IL18.	[23]
miR-341	Experimental (rats)	Sciatic nerve	Model of chronic bilateral sciatic nerve compression	Overexpression of miR-341 induces the development of CBP.	[37]
miR-378	Experimental (rats)	Spinal cord (neurons)	Model of chronic sciatic nerve injury; models of mechanical and thermal hyperalgesia	Overexpression of miR-378 inhibits CBP by targeting EZH2.	[56]
miR-381	Experimental (rats)	Sciatic nerve	Model of chronic sciatic nerve injury	Overexpression of miR-381 inhibits the development of neuropathic pain by targeting HMGB1 and CXCR4.	[57]
miR-429	Experimental (mice)	Sciatic nerve	Model of partial unilateral ligation of the sciatic nerve	Hypo-expression of miR-429 induces DNMT3a production, which is involved in generating dysfunction of the mesolimbic motivation/evaluation circuitry that links prolonged nociceptive stimuli with comorbidities in CBP (anxiety and sleep disorders).	[73]
	Experimental (rats)	Spinal cord (neurons). Isolated microglial cells	Model of chronic damage and compression of spinal roots	Overexpression of miR-429 inhibits the production of proinflammatory cytokines (IL6, IL1β, and TNFα) by targeting ZEB1.	[54]
miR-431	Experimental (mice)	Dorsal spinal root ganglia (neurons)	Model of partial unilateral ligation of the sciatic nerve	Overexpression of miR-431 induces IL-6 release, facilitating the development of neuropathic pain.	[25]
miR -500	Experimental (rats)	Spinal cord (neurons of the dorsal horn). Posterior spinal roots (neurons)	Model of peripheral neuropathy (anterior root L5) induced by paclitaxel	Overexpression of miR-500 induces the development of neuropathic pain and regulates GAD67 levels. Hypo-expression of miR-500 is associated with a deficit of GABAergic synapses and a weakening of sensitized pain behavior.	[74]
miR-511-3p	Experimental (mice)	Spinal cord (neurons of the dorsal horn)	Model of partial unilateral ligation of the sciatic nerve	Overexpression of miR-511-3p induces IL-6 release, facilitating the development of neuropathic pain.	[25]
miR-541-3p	Experimental (rats)	Sciatic nerve	Model of chronic bilateral compression of the sciatic nerves	Hypo-expression of miR-541-3p induces the development of CBP.	[37]
miR-let-7a	Experimental (rats)	Spinal cord (neurons of the dorsal horn)	Model of damage by local induction by an adeno-associated virus vector	Hypo-expression of miR-let-7a induces the expression of the β2 sodium channel subunit protein, which leads to long-term hyperactivity of damaged neurons and the development of CBP	[75]
miR-let-7b	Experimental (mice)	Posterior spinal root ganglia (neurons)	Model of formaldehyde-induced injury	Overexpression of miR-let-7b induces inward ion currents through interaction between TLR-7 and nociceptive ion receptor subfamily cation channels, which induces and maintains CBP.	[76]
Circulating microRNA					
miR-30c-5p	Experimental (anti-allodyn phenotype of mice)	Blood (plasma)	Chronic sciatic nerve injury model	Overexpression of miR-30c-5p induces the development of neuropathic pain via BAMBI (TGFβ pseudo-receptor).	[28]
miR-124	Experimental (mice)	Blood (serum, macrophages)	IL-1β-induced inflammatory hyperalgesia model in mice with chronic carrageenan-induced hyperalgesia	Overexpression of miR-124 shifts the “pro-inflammatory M1/anti-inflammatory M2” balance toward the anti-inflammatory M2 phenotype and reduces mechanical hyperalgesia and pain behavior.	[80]
miR-221	Experimental (rats)	Blood (serum, serum exosomes)	Model of diabetic neuropathic pain by administration of streptozotocin	Overexpression of miR-221 inhibits pain associated with proinflammatory mediators (bradykinin, PGE2, IL6, IL1β, and TNFα) by targeting SOCS3.	[36]
miR-590-3p	Experimental (mice)	Blood (serum, plasma, T cells)	Model of diabetic neuropathic pain	Overexpression of miR-590-3p inhibits T-cell infiltration by targeting RAP1A, thereby reducing neuropathic pain.	[36]

Note: AC-9—antinuclear factor; ADAMATS—disintegration and metalloproteinase with thrombospondin motif type 1; AMPA—α-amino-3-hydroxy-5-methyl-4-isoxazolepropionic acid receptor; AMPK—5′-adenosine monophosphate (AMP)-activated protein kinase; AKT—protein kinase B; ATF—activating transcription factor; ASC—atypical squamous epithelial cells; BACE1—beta-site amyloid precursor protein-cleaving enzyme type 1; BALB/c mice—albino house mouse; BAMBI—BMP and activin membrane-bound inhibitor homolog; BDNF—brain-derived neurotrophic factor; cAMP—cyclic adenosine monophosphate; CBP—chronic pain syndrome; CCL2—cytokine, belongs to the group of CC-chemokines; CCR1—chemokine receptor type 1; CEBPB—CCAAT/enhancer-binding protein beta; Circarl15—circular RNA molecule; COX—cyclooxygenases; CREB—transcription factor capable of binding CRE-sequences of DNA; CTNNB1—catenin beta-1 gene; CXCL13- chemokine ligand 13; CXCR4—chemokine receptor type 4; CX43—connexin 43; DISC1—DISC1 scaffold protein; DNMT3a—methyltransferase 3A; ECM—extracellular matrix; ERK—extracellular signal-regulated kinases; EZH2—enhancer of zeste homolog 2; JNK—Janus kinase; GABA—gamma-aminobutyric acid; GAD67—glutamate decarboxylase; GCSF—granulocyte colony-stimulating factor; GDF5—growth/differentiation factor 5; GluA—ionotropic glutamate receptors; GSK3B—glycogen synthase kinase-3 beta; HIF-1α—hypoxia-inducible factor 1-alpha; HMGB1—high mobility group box 1 protein; IL—interleukin; iNOS—inducible NO synthase; IRAK1—IL-1 receptor-associated kinase 1; IVDD—intervertebral disc degeneration; IVD—intervertebral disc; LPS—lipopolysaccharide; MAPK—membrane-activated protein kinase; MeCP2—methyl-CpG-binding protein 2; MIP—macrophage inflammatory proteins; miR—microRNA; MMP—matrix metalloproteinase; mTOR—mammalian target of rapamycin; MyD88—primary myeloid differentiation response 88; mRNA—matrix ribonucleic acid; NF-κB—nuclear factor kappa B; NLRP3—NOD-like receptor pyrin domain containing 3; NOX4—NADPH oxidase 4; NR2A—NMDA receptor 2A; NP—nucleus pulposus; P-Creb—phosphorylation of cyclase response element binding protein; PDK4—pyruvate dehydrogenase kinase, isozyme 4; PGE2—prostaglandin E2; PTEN—phosphatase and tensin homolog; p65—transcription factor p65; RAP1A—Ras-associated protein 1A; SG—stress granule protein; SDF-1—stromal cell-derived factor-1; SLC17A6—solute carrier family 17 (vesicular glutamate transporter); SOCS3—3′-UTR suppressor of cytokine signaling 3; SOX9—SRY-box transcription factor 9; STAT—activator of transcription; TGF-β—transforming growth factor beta; TIA-1—T-cell antigen; TIR—Toll/IL-1 signaling pathway; TLR—toll-like receptor; TNF—tumor necrosis factor; TRAF6—cytosolic adapter protein belonging to the TNF receptor-associated factor family; TXNIP—thioredoxin-interacting protein; VEGF—vascular endothelial growth factor; ZEB1—zinc finger E-box-binding protein 1.

### 3.2. Clinical Studies

Clinical studies have been conducted primarily among patients with IVDD with and without lumbar IVD hernias [84,85,86]. The control group in several studies included individuals with idiopathic scoliosis [31,87,88] and lumbar vertebral fractures [89,90,91]. The biospecimens for studying the expression level of *Homo sapiens* miR (hsa-miR) were blood serum and IVD surgical material after lumbar discectomy. A mandatory condition for the study was magnetic resonance imaging of the lumbosacral spine to objectively confirm the diagnosis of IVDD, as well as laboratory tests to determine the level of leukocytes, leukocyte formula, C-reactive protein level, and other biomarkers of the systemic inflammatory response. According to the clinical studies presented in Table 2, the most studied hsa-miRs associated with the activation and maintenance of the systemic inflammatory response in patients with IVDD are hsa-miR-7; hsa-miR-15a; hsa-miR-20a; hsa-miR-21; hsa-miR-23c; hsa-miR-27a; hsa-miR-30d; hsa-miR-34a; hsa-miR-96; hsa-miR-129-5p; hsa-miR-132; hsa-miR-141; hsa-miR-143; hsa-miR-146a; hsa-miR-148a; hsa-miR-149-5p; hsa-miR-154; hsa-miR-184; hsa-miR-210; hsa-miR-221; hsa-miR-222; hsa-miR-222-3p; hsa-miR-328-5p; hsa-miR-365; hsa-miR-431-5p; hsa-miR-494; hsa-miR-532; hsa-miR-625-5p; hsa-miR-640; hsa-miR-654-5p; and hsa-miR-3150a-3p [31,39,42,58,80,84,86,87,88,92,93,94,95,96,97,98,99,100,101,102,103,104,105,106,107,108,109,110,111,112,113,114].

The most studied hsa-miRs possessing protective properties in relation to the systemic inflammatory response were the following: hsa-miR-15a, hsa-miR-15A-5p, hsa-miR-15b, hsa-miR-27b; hsa-miR-31-5p; hsa-miR-93; hsa-miR-96; hsa-miR-98; hsa-miR-129-5p; hsa-miR-133a; hsa-miR-140-5p; hsa-miR-145; hsa-miR-146a; hsa-miR-148a; hsa-miR-155; hsa-miR-155-3p; hsa-miR-155-5p; hsa-miR-184; hsa-miR-193-3p; hsa-miR-202-3p; hsa-miR-365; hsa-miR-455-5p; hsa-miR-483-3p; hsa-miR-486-5p; hsa-miR-494; hsa-miR-558; hsa-miR-573; hsa-miR-625; hsa-miR-660; and hsa-miR-665 [17,33,39,58,60,85,89,90,91,92,96,97,100,105,115,116,117,118,119,120,121,122,123,124,125,126,127,128,129,130,131,132].

These hsa-miRs, due to the phenomenon of peripheral and central sensitization, modulate the increase or decrease in CBP in patients with IVDD depending on their proinflammatory or anti-inflammatory effects, which was previously demonstrated in animal models (Table 1).

Of particular note are the hsa-miRs involved in the processes of facilitating the regulation of genes responsible for the formation of CBP in patients with IVDD: hsa-miR-23c; hsa-miR-124a; hsa-miR-132-3p; hsa-miR-149-5p; hsa-miR-155; hsa-miR-194-5p; and hsa-miR-365 [39,71,103,111,117,133], which may be used to develop new therapeutic strategies in the future.

**Table 2:** Clinical studies of the role of microRNA in chronic back pain development in patients with intervertebral disc degeneration.

MicroRNA	Design of the Study	Tissue (Sample)	General Characteristics of the Sample	Effects of the microRNA	References
A. IVDD-specific model of chronic pain					
Local microRNA					
hsa-miR-7	Clinical (humans and cell culture)	IVD tissues (surgical material)	Patients with IVDD (main group = 12; age range—20–42 years; mean age = 27.4 years) and patients with idiopathic scoliosis (control group = 8; age range = 18–40 years; mean age = 21.8 years).	Overexpression of hsa-miR-7, targeting IL1β, induces ECM degradation in IVD via targeting GDF5.	[134]
hsa-miR-15a	Clinical (humans)	NP tissues (surgical material)	Patients with IVDD (main group = 20; age range = 18–24 years; mean age = 25.4 years) and patients with idiopathic scoliosis (control group = ND; age = ND).	Overexpression of hsa-miR-15a inhibits NP cell proliferation and induces cell apoptosis by targeting MAP3K9.	[97]
hsa-miR-15a-5p	Clinical (humans)	NP tissues (surgical material)	Patients with IVDD (main group = ND; age = ND) and patients with lumbar spine fractures (control group = ND; age = ND).	Overexpression of hsa-miR-15A-5p inhibits NP cell proliferation, induces SOX9 expression to suppress inflammatory response and apoptosis via NF-Κb signaling pathway.	[58]
hsa-miR-15b	Clinical (humans)	NP tissues (surgical material)	Patients with IVDD (main group = 15; age range = 29–62 years; mean age = 36.2 years) and patients with idiopathic scoliosis (control group = 10; age range = 20–36 years; mean age = 24.3 years).	Hypo-expression of hsa-miR-15b inhibits ECM degradation in NP cells via increased SMAD3 expression.	[120]
hsa-miR-20a	Clinical (humans)	IVD cartilage end plate (surgical material)	Adult patients with IVDD (48 participants; age range = 14–71 years; mean age = ND).	Overexpression of hsa-miR-20a inhibits ANKH, which is associated with inorganic phosphate-induced calcification and IVDD progression.	[93]
hsa-miR-21	Clinical (humans)	NP tissues (surgical material)	Adult patients with IVDD (main group = 65; age range = 45–67 years; mean age = 54.6 years) and normal IVD (control group = 45; age range = 17–22 years; mean age = 20.4 years).	Overexpression of hsa-miR-21 induces the Akt/mTOR pathway by suppressing PTEN, leading to overexpression of MMP3 and MMP9 and subsequent ECM degradation in degenerating IVDs.	[86]
hsa-miR-23c	Clinical (humans)	NP tissues (surgical material)	Patients with IVDD (main group = 24; age = ND) and patients with spinal cord injury (control group = 16; age = ND).	Overexpression of hsa-miR-23c and CTNNB1 induces the development of IVDD.	[39]
hsa-miR-27a	Clinical (humans)	NP tissues (surgical material)	Patients with lumbar disc herniation (main group = 20; age range = ND; mean age = 54.9 ± 7.5 years) and patients with spinal cord injury (control group = 20; age range = ND; mean age = 42.2 ± 8.9 years).	Overexpression of hsa-miR-27a induces the release of proinflammatory mediators in degenerating IVD cells via the MAPK signaling pathway.	[98]
hsa-miR-27b	Clinical (humans)	NP tissues (surgical material)	Adult patients with IVDD (main group = 80; age range—48–69 years; mean age = 56.2 years) and adult patients with lumbar vertebral fractures (control group = 76; age range = 18–23 years; mean age = 20.2 years).	Hypo-expression of hsa-miR-27b induces MMP13 production in IVDD.	[89]
hsa-miR-30d	Clinical (humans)	NP tissues (surgical material)	Patients with IVDD (main group = 20; age = ND) and patients with idiopathic scoliosis (control group = 10; age = ND).	Overexpression of hsa-miR-30d induces apoptosis and ECM degradation by upregulating SOX9 in NPs of degenerating IVDs.	[99]
hsa-miR-31-5p	Clinical (humans)	NP tissues (surgical material)	Patients with IVDD (main group = 82; age range = ND; mean age = 57.6 ± 5.3 years) and patients with traumatic fractures of the lumbar vertebrae (control group = 91; age range = ND; mean age = ND).	Overexpression of hsa-miR-31-5p induces NP cell proliferation, inhibits apoptosis, promotes ECM formation by regulating the SDF-1/CXCR7 pathway.	[60]
hsa-miR-34a	Clinical (humans)	IVD cartilaginous endplate tissue (surgical specimen)	Adult patients with IVDD (main group = 12; age range = 61–75 years; mean age = 69 years) and adult patients with idiopathic scoliosis (control group = 4; age range = 19–25 years; mean age = 21 years).	Overexpression of hsa-miR-34a inhibits production of the apoptosis regulator BCL2.	[87]
hsa-miR-93	Clinical (humans)	IVD tissues (surgical material)	Patients with IVDD (main group = 54; age range = 29–64 years; mean age = 48.2 years) and patients with idiopathic scoliosis (control group = 4; age range = 16–20 years; mean age = 21 years).	Hypo-expression of hsa-miR-93 induces MMP3 production and degradation of type II Col in IVD NP.	[115]
hsa-miR-96	Clinical (humans)	NP tissues (surgical material)	Patients with IVDD (main group = 30; age range = ND; mean age = 50 ± 11.7 years) and patients with traumatic lumbar spine fracture (control group = 5; age range = ND; mean age = 21.6 ± 3.8 years).	Overexpression of hsa-miR-96 induces NP cell proliferation by targeting the activation of the AKT signaling pathway.	[100]
hsa-miR-98	Clinical (humans)	IVD tissues (surgical material)	Patients with IVDD (main group = 116; age range = 47–72 years; mean age = 58.2 years) and patients with recent lumbar vertebral fractures (control group = 102; age range = 18–22 years; mean age = 20.3 years).	Overexpression of hsa-miR-98 targets signaling enzymes involved in ECM metabolism and the IL-6/STAT3 signaling pathway, which prevents the development of IVDD and CBP.	[92]
hsa-miR-129-5p	Clinical (humans)	NP tissues (surgical material)	Patients with IVDD (main group = 33; age range = 32–56 years; mean age = 45.4 years) and patients with idiopathic scoliosis (control group = 29; age range = 18–24 years; mean age = 21.1 years).	Hypo-expression of hsa-miR-129-5p induces the development of IVDD via inhibition of apoptosis by targeting BMP2.	[101]
	Clinical (humans)	NP tissues (surgical material)	Patients with IVDD (main group = 30; age range = 26–55 years; mean age = 41.7 years) and patients with idiopathic scoliosis (control group = 30; age range = 20–66 years; mean age = 42.4 years).	Overexpression of hsa-miR-129-5p inhibits the p38-mapk pathway by targeting leucine-rich α2-glycoprotein 1, thereby exerting a protective effect on the development of IVDD.	[121]
hsa-miR-132	Clinical (humans)	NP tissues (surgical material)	Adult patients with IVDD (main group = 27; age range = 29–70 years; mean age = 44 years) and adult patients with idiopathic scoliosis (control group = 14; age range = 17–34 years; mean age = 20.5 years).	Overexpression of hsa-miR-132 induces ECM degradation by directly targeting GDF5 and leading to increased expression of MMP13 and ADAMTS4 via the MAPK/ERK pathway.	[31]
hsa-miR-138-5p	Clinical (humans)	NP tissues (surgical material)	Patients with IVDD (main group = 23; age range = 18–39 years; mean age = 23.4 years) and adult patients with idiopathic scoliosis (control group = ND; age = ND).	Overexpression of hsa-miR-138-5p induces NP cell apoptosis by regulating PTEN/PI3K/Akt signaling or by directly acting through sirtuin 1; hypo-expression of hsa-miR-138-5p inhibits TNFα-induced apoptosis in NP cells through the PTEN/PI3K/Akt signaling pathway.	[135]
hsa-miR-140-5p	Clinical (humans)	NP tissues (surgical material)	Patients with IVDD (22 participants; age range = 33–78 years; mean age = 53 years).	Overexpression of hsa-miR-140 inhibits the local inflammatory response and IVD degeneration by reducing the production of proinflammatory cytokines and increasing the production of aggrecan and Col II; inhibits TLR4 expression.	[122]
hsa-miR-141	Clinical (humans)	NP tissues (surgical material)	Patients with IVDD (main group = 82; age range = 18–39 years; mean age = 23.4 years) and patients with recent traumatic lumbar fractures (control group = 68; age range = 18–39 years, mean age = 23.4 years).	Overexpression of hsa-miR-141 induces ECM degradation by targeting and depleting SIRT1 (a negative regulator of the NF-κB pathway).	[94]
hsa-miR-143	Clinical (humans)	NP tissues (surgical material)	Patients with IVDD and herniation at the lumbar spine level (main group = 10; age range = 28–58 years, mean age = 25.8 years) and adult patients with idiopathic scoliosis (control group = 10; age range = 17–40 years; mean age = 22.3 years).	Overexpression of hsa-miR-143 induces NP cell apoptosis through direct action and inhibition of BCL2 (an enzyme that blocks apoptosis).	[95]
hsa-miR-145	Clinical (humans)	IVD tissues (surgical material)	Adult patients with IVDD (main group = ND; age = ND) and adult patients with thoracolumbar vertebral fractures (control group = ND; age = ND).	Overexpression of hsa-miR-145 targeting Adamats17 inhibits apoptosis of degenerating IVD cells in vitro, both in the presence and absence of oxidative stress.	[90]
hsa-miR-146a	Clinical (humans)	NP tissues (surgical material)	Patients with IVDD and herniation at the lumbosacral spine level (main group = 5; age range = 50–70 years; mean age = 57.5 years) and patients with lumbar spine fracture (control group = 5; age range = 52–69 years; mean age = 56.6 years).	Overexpression of hsa-miR-146a induces the development of IVDD and a local inflammatory response by increasing IL6 production and activating the STAT3 signaling pathway.	[102]
hsa-miR-148a	Clinical (humans)	NP tissues (surgical material)	Patients with IVDD (main group = 30; age = ND) and healthy volunteers (control group = 30; age = ND).	Overexpression of hsa-miR-148a inhibits the production of proinflammatory mediators via the P38-MAPK pathway, regulating the development and progression of IVDD.	[124]
	Clinical (humans)	NP tissues (surgical material)	Patients with IVDD and herniation at the lumbosacral spine level (main group = 5; age range = 45–58 years; mean age = ND) and patients with lumbar spine fracture or idiopathic scoliosis (control group = 5; age range = 18–20 years; mean age = ND).	Overexpression of hsa-miR-148a induces increased Hotair activity, which in turn increases PTEN expression and leads to increased severity of IVDD.	[41]
hsa-miR-149-5P	Clinical (humans)	NP tissues (surgical material)	Patients with IVDD (main group = 40; age range = ND; mean age = 24.3 ± 5.1 years) and healthy volunteers or patients with idiopathic scoliosis (control group = 40; age range = ND; mean age = 22.4 ± 4.2 years).	Overexpression of hsa-miR-149-5P activates LINC00917; inhibits NP cell proliferation; initiates inflammation and pyroptosis by regulating the pyrin domain containing 1 signaling pathway of the miR-149-5P/NLR family.	[103]
hsa-miR-154	Clinical (humans)	NP tissues (surgical material)	Patients with IVDD (main group = 8; age range = 19–38 years; mean age = 23.1 years) and patients with idiopathic scoliosis (control group = 8; age range = 17–42 years; mean age = 28.3 years).	Overexpression of hsa-miR-154 induces IVDD development by targeting FGF14.	[104]
hsa-miR-155	Clinical (humans and tissue culture)	Synovial fibroblasts and synovial tissue	Adult patients with inflammatory tissue injury IVD (participants = ND; age = ND).	Overexpression of hsa-miR-155 inhibits MMP3 production and reduces the effects of MMP3 and MMP1 on TLR ligands and cytokines.	[136]
	Clinical (humans and tissue culture)	NP cells (surgical material)	Adult patients with inflammatory IVD tissue injury (participants = ND; age = ND).	Overexpression of hsa-miR-155 inhibits ECM degradation via p65/NF-Κb signaling.	[33]
	Clinical (humans)	NP tissues (surgical material)	Patients with IVDD (main group = 3; age range = 65–70 years; mean age = ND) and patients with idiopathic scoliosis (control group = 3; age range = 13–15 years; mean age = ND).	Hypo-expression of hsa-miR-155 inhibits the expression of Col II and glycosaminoglycans by targeting ERK1/2.	[125]
hsa-miR-155-3p	Clinical (humans)	NP tissues (surgical material)	Patients with IVDD (main group = 36; age range = 26–62 years, mean age = 39.43 years) and patients with traumatic lumbar spine fracture (control group = 31; age range = 12–24 years, mean age = 14.76 years).	Overexpression of hsa-miR-155-3p inhibits the expression of KDM3A and HIF1α.	[126]
hsa-miR-155-5p	Clinical (humans)	NP and AF tissues (surgical material)	Patients with IVDD and lumbar disc herniation (participants = 6; age = ND).	Overexpression of hsa-miR-155-5p induces the production of proinflammatory cytokines (IL8, IL6); induces BDNF expression; and activates MAPK through increased phosphorylation of p38 and p53. Hypo-expression of hsa-miR-155-5p induces the anti-inflammatory cytokines IL10 and TIMP4.	[127]
hsa-miR-184	Clinical (humans)	NP tissues (surgical material)	Patients with IVDD (main group = 40; age range = 29–57 years; mean age = ND) and adolescents with idiopathic scoliosis (control group = 4; age range = 11–14 years; mean age = ND).	Overexpression of hsa-miR-184 induces IVD cell proliferation via regulation of GAS1, which induces Akt phosphorylation.	[105]
hsa-miR-193-3p	Clinical (humans and tissue culture)	NP tissues (surgical material)	Adult patients with IVDD (main group = 128; age range = 45–69 years; mean age = 53.9 years) and patients with recent traumatic lumbar spine fracture (control group = 116; age range = 18–22 years, mean age = 20.6 years).	Overexpression of hsa-miR-193a-3p inhibits IVDD progression in vitro and in vivo. Hypo-expression of has-miR-193a-3p induces MMP14 expression and IVD degradation.	[118]
hsa-miR-194-5p	Clinical (humans)	NP tissues (surgical material).	Patients with IVDD (main group = 6; age = ND) and healthy volunteers (control group = 3; age = ND).	Hypo-expression of hsa-miR-194-5p inhibits Cullin family genes (*CUL4A* and *CUL4B*).	[133]
hsa-miR-202-3p	Clinical (humans)	NP tissues (surgical material)	Patients with IVDD (participants = 40; age range = 23–82 years, mean age = 55.1 years).	Overexpression of hsa-miR-202-3p inhibits IL-1β-induced MMP1 expression. Transfection of cells with an hsa-miR-202-3p inhibitor significantly increases MMP1.	[128]
hsa-miR-210	Clinical (humans)	NP tissues (surgical material)	Patients with IVDD (main group = 45; age range = 38–65 years, mean age = 52.7 years) and patients with traumatic lumbar spine fracture (control group = 5; age range = 16–20 years; mean age = 18.6 years).	Overexpression of hsa-miR-210 induces the development of IVDD; it directly targets ATG7 and then prevents autophagy, leading to increased expression of MMP3 and MMP13 and subsequent degradation of Col II and ECM aggrecan.	[106]
hsa-miR-221	Clinical (humans)	NP tissues (surgical material)	Patients with IVDD (main group = 15, age range = ND; mean age = 48 ± 7 years) and patients with idiopathic scoliosis (control group = 3; age range = ND; mean age = 38 ± 11 years).	Overexpression of hsa-miR-221 targets ERα, which influences the protective effect of estrogen on the development of IVDD.	[107]
hsa-miR-222	Clinical (humans)	NP tissues (surgical material)	Patients with IVDD (main group = 22; age range = 26–62 years; mean age = 39.4 years) and patients with traumatic lumbar spine fracture (control group = 9; age range = 12–24 years; mean age = 14.76 years).	Overexpression of hsa-miR-222 induces local inflammatory response and apoptosis in degenerating IVDs by targeting TIMP3 miR.	[108]
hsa-miR-222-3p	Clinical (humans)	NP tissues (surgical material)	Patients with IVDD (main group = 30; age = ND) and patients with lumbar spine fractures (control group = 10; age = ND).	Overexpression of hsa-miR-222-3p induces IVDD development by targeting CdKn1b.	[109]
hsa-miR-328-5p	Clinical (humans)	NP tissues (surgical material)	Patients with IVDD (main group = 10; age range = ND; mean age = 41.6 ± 4.8 years) and healthy volunteers (control group = 10; age range = ND; mean age = 38.5 ± 3.5 years).	Overexpression of hsa-miR-328-5p inhibits proliferation and induces apoptosis of NP cells by regulating the expression of Bcl2, Bax, and caspase-3, and promotes the progression of premature aging IVD.	[110]
hsa-miR-365	Clinical (humans and tissue culture)	IVD cartilage end plate (surgical material)	Patients with IVDD (main group = 38; age range = ND; mean age = 58 years) and adult patients with fractures and dislocations of the cervical spine (control group = 20; age range = ND; mean age = 34 years).	Overexpression of hsa-miR-365 targets HDAC4, promoting tissue protection in IVDD.	[119]
	Clinical (humans)	NP tissues (surgical material)	Patients with IVDD and herniation at the lumbar level (main group = 10; age range = 45–65 years, mean age = 52 ± 10 years) and patients with traumatic lumbar spine fracture (control group = 10; age range = 45–65 years, mean age = 53 ± 11 years).	Overexpression of hsa-miR-365 and MT1DP and hypo-expression of NRF2 are associated with IVDD, apoptosis, and the development of a local inflammatory response.	[111]
hsa-miR-431-5p	Analysis of the *GSE67567* gene expression dataset; clinical (humans)	Database Omnibus NP tissues (surgical material)	Patients with IVDD (main group = 5; age = ND) and volunteers with Hirayama disease (control group = 5; age = ND).	Overexpression of hsa-miR-431-5p inhibits Circarl15 production in IDD tissues. Circarl15 expression positively correlates with the DISC1 scaffold protein. Circarl15 suppresses NP cell apoptosis but promotes NP cell proliferation through regulation of the hsa-miR-431-5p/disc1 signaling axis.	[42]
hsa-miR-455-5p	Clinical (humans and tissue culture)	IVD cartilage end plate (surgical material)	Adult patients with IVDD (45 participants; age range = ND; mean age = 58.67 ± 10.02).	Overexpression of hsa-miR-455-5p inhibits the development of IVDD via the TGF-β/SMAD signaling pathway by regulating RUNX2.	[85]
hsa-miR-483-3p	Clinical (humans)	NP tissues (surgical material)	Adult patients with IVDD (main group = 24; age = ND) and adult patients with spinal cord injury (control group = 16; age = ND).	Hypo-expression of hsa-miR483-3p and GSK3B induces the development of IVDD.	[39]
hsa-miR-486-5p	Clinical (humans)	NP tissues (surgical material)	Adult patients with IVDD (20; age range = 18–38 years, mean age = 21.4 years).	Overexpression of hsa-miR-486-5p increases NP cell viability; inhibits proinflammatory cytokines and ECM degradation; and partially inhibits FoxO1 expression.	[129]
hsa-miR-494	Clinical (humans)	NP tissues (surgical material)	Patients with IVDD and herniation at different levels (main group = 29; age range = 15 to 76 years, mean age = 20.6 years) and IVD tissue samples from healthy volunteers (control group = 8; age range = from 1 month to 10 years; mean age = ND).	Overexpression of hsa-miR-494 activates SOX9 and prevents the development of IVDD.	[137]
	Clinical (humans)	NP tissues (surgical material)	Patients with IVDD (20 participants; age range = 30–58 years, mean age = 46.5 years).	Overexpression of hsa-miR-494 inhibits ECM degradation; inhibits MMP, disintegrin, and ADAMTS activity by directly targeting SOX9.	[96]
hsa-miR-573	Clinical (humans)	NP cells (surgical material)	Patients with IVDD (main group = 30; age—ND) and patients with idiopathic scoliosis (control group = 30, age—ND).	Overexpression of hsa-miR-573 enhances NP cell viability and inhibits apoptosis in IVDD via targeting BAX.	[130]
hsa-miR-625	Clinical (humans)	NP cells (surgical material)	Patients with IVDD of the cervical spine (main group = 6; age range = 46–62 years, mean age = 52.6 years) and patients with fracture of the cervical spine (control group = 3; age range = 38–58 years; mean age = 47.2 years).	Hypo-expression of hsa-miR-625 induces Fas-mediated apoptosis; inhibits Bcl2 expression.	[133]
hsa-miR-625-5p	Clinical (humans and cell culture)	NP and AF cell culture (LPS-induced inflammation model)	Adult patients with IVDD (main group = 72; age range = ND; mean age = 45.3 ± 3.9 years) and healthy volunteers (control group = 24; age range = ND; mean age = 41.7 ± 4.1 years).	Overexpression of hsa-miR-625-5p directly induces damage to the ECM structural protein (Col type I) via NF-kB and TLR4 signaling pathway after LPS stimulation.	[84]
hsa-miR-640	Clinical (humans)	NP cells (surgical material)	Patients with IVDD (main group = 15; age range = 27–46 years; mean age = 34.6 years) and patients with idiopathic scoliosis (control group = 8; age range = 18–33 years, mean age = 24.13 years).	Overexpression of hsa-miR-640 induces IVDD; induces apoptosis of IVD cells; induces MMP3 and MMP9 production; inhibits aggrecan and Col type II through the NF-κB signaling pathway.	[88]
hsa-miR-654-5p	Clinical (humans)	NP cells (surgical material)	Patients with IVDD (main group = 54; age range = 31–53 years, mean age = 42.3 years) and healthy volunteers (control group = 4; age range = 24–45 years, mean age = 33.6 years).	Overexpression of hsa-miR-654-5p induces the development of IVDD; induces the production of MMP3, MMP9, and MMP13; inhibits autophagy via the PI3K/AKT/mTOR pathway.	[115]
hsa-miR-660	Clinical (humans)	NP cells (surgical material)	Adult patients with IVD hernia (main group = 3, age range = ND; mean age = 39 ± 10 years) and adult patients with lumbar vertebral fracture (control group = 3; age range = ND; mean age = 36 ± 7 years).	Hypo-expression of hsa-miR-660 inhibits NP cell apoptosis associated with downregulation of C-caspase 3 and C-caspase 7.	[91]
hsa-miR-665	Clinical (humans)	NP cells (surgical material)	Adult patients with IVDD (main group = 35; age = ND) and adult patients with idiopathic scoliosis (control group = 5; age = ND).	Overexpression of hsa-miR-665 induces NP cell proliferation; inhibits the expression of type II Col and aggrecan; induces the expression of MMP3 and MMP13; inhibits the expression of GDF 5.	[132]
hsa-miR-3150a-3p	Clinical (humans)	NP cells (surgical material)	Adult patients with IVDD (main group = 20; age range = ND; mean age = 41.15 years) and adult patients with lumbar spine fractures (control group = 20; age range = ND; mean age = 37.75 years).	Overexpression of hsa-miR-3150a-3p induces IVDD by targeting aggrecan in the ECM.	[114]
Circulating microRNA					
hsa-miR-133a	Clinical (humans)	Blood (plasma)	Patients with IVDD (matched group = 45; age range = ND; mean age = 58.4 ± 8.3 years) and healthy volunteers (control group = 53; age range = ND; mean age = 55.1 ± 7.5 years).	Hypo-expression of hsa-miR-133a induces degradation of type II Col via overexpression of MMP9.	[116]
hsa-miR-146a	Clinical (humans)	Blood (mononuclear cells)	Adult patients with inflammatory IVD tissue injury (21 participants; age range = 33–73 years; mean age = ND).	Overexpression of hsa-miR-146a induces a systemic inflammatory response.	[138]
	Clinical (humans)	Blood (plasma)	Patients with IVDD and lumbosacral disc herniation (main group = 21; age = ND) and healthy volunteers (control group = 21; age = ND).	Overexpression of hsa-miR-146a inhibits the production of proinflammatory cytokines (IL1β, IL6, and TNFα) by targeting the TARF6/NF-Κb pathway.	[123]
hsa-miR-148a	Clinical (humans)	Blood (plasma).	Patients with IVDD (main group = 30; age = ND) and healthy volunteers (control group = 30; age = ND).	Overexpression of hsa-miR-148a inhibits the production of proinflammatory mediators via the P38-MAPK pathway, regulating the development and progression of IVDD.	[124]
hsa-miR-155	Clinical (humans and tissue culture)	Blood (mononuclear cells)	Adult patients with inflammatory tissue injury IVD (participants = ND; age = ND).	Overexpression of hsa-miR-155 inhibits MMP3 production and reduces the effects of MMP3 and MMP1 on TLR ligands and cytokines.	[136]
hsa-miR-194-5p	Clinical (humans)	Blood (plasma)	Patients with IVDD (main group = 144; age = ND) and healthy volunteers (control group = 24; age = ND).	Hypo-expression of hsa-miR-194-5p inhibits Cullin family genes (*CUL4A* and *CUL4B*).	[133]
hsa-miR-532	Clinical (humans)	Blood (plasma) NP tissues (surgical material)	Patients with IVDD (main group = 20; age = ND) and healthy volunteers (control group = 20; age = ND).	Overexpression of hsa-miR-532 induces apoptosis in NP cells, induces the development of IVDD by targeting Bcl9, and inhibits the Wnt/β-catenin pathway.	[112]
hsa-miR-625-5p	Clinical (humans and cell culture)	Blood (serum and plasma)	Adult patients with IVDD (main group = 72; age range = ND; mean age = 45.3 ± 3.9 years) and healthy volunteers (control group = 24; age range = ND; mean age = 41.7 ± 4.1 years).	Overexpression of hsa-miR-625-5p directly induces damage to the ECM structural protein (Col type I) via NF-kB and TLR4 signaling pathway after LPS stimulation.	[84]
B. Non-IVDD-specific model of chronic pain					
Local microRNA					
hsa-miR-146a	Clinical (humans)	Articular cartilage and synovial membrane (surgical material)	Adult patients with osteoarthritis of the knee (participants = ND; age = ND).	Overexpression of hsa-miR-146a induces a local inflammatory response, leading to the production of proinflammatory mediators (TNFα, COX2, iNOS and IL-6).	[80]
hsa-miR-558	Clinical (humans)	Knee cartilage (surgical material)	Adult patients with osteoarthritis of the knee joints (main group = 20; age range = ND; mean age = 71.23 ± 7.10 years) and healthy adult volunteers (control group = 20; age range = ND; mean age = 72.1 2 ± 9.78 years).	Overexpression of hsa-miR-558 inhibits IL-1β-induced COX2 activation, which promotes systemic inflammatory response.	[17]
Circulating microRNA					
hsa-miR-124a	Clinical (humans)	Blood (primary T cells)	Adult patients with neuropathic pain associated with polyneuropathy, post-traumatic neuralgia, trigeminal neuralgia/neuropathy (main group = 11; age range = ND; mean age = 54 ± 12 years), and healthy volunteers (control group = 9; age range = ND; mean age = 36 ± 9 years).	Overexpression of hsa-miR-124a inhibits SIRT1 production and enhances neuropathic pain.	[117]
hsa-miR-132-3p	Clinical (humans)	Blood. Biopsy material of sural nerves	Adult patients with non-inflammatory and inflammatory neuropathy of different origins (main group = 55; age range = 33–84 years; mean age = 66 years), and healthy volunteers (control group = 30; age range = 38–69 years; mean age = 56.5 years).	Overexpression of hsa-miR-132-3p induces the development of neuropathic pain and CBP by upregulating the AMPA receptor subunits GluA1 and GluA2 in the spinal cord.	[71]
hsa-miR-155	Clinical (humans)	Blood (primary T cells)	Adult patients with neuropathic pain associated with polyneuropathy, post-traumatic neuralgia, trigeminal neuralgia/neuropathy (main group = 11; age range = ND; mean age = 54 ± 12 years), and healthy volunteers (control group = 9; age range = ND; mean age = 36 ± 9 years).	Overexpression of hsa-miR-155 inhibits SIRT1 production, increasing neuropathic pain.	[117]

Note: ADAMATS—thrombospondin motif-associated metalloproteinase; AKT—protein kinase B; AMPA—α-amino-3-hydroxy-5-methyl-4-isoxazolepropionic acid receptor; ANKH—progressive ankylosis protein homolog; ATG7—autophagy-related 7; BAX—apoptosis regulator; BCL—intracellular B-cell lymphoma; BDNF—brain-derived neurotrophic factor; BMP2—bone morphogenetic protein 2; CBP—chronic pain syndrome; CdKn1b—cyclin-dependent kinase inhibitor 1B; Circarl15—circular RNA molecule; Col—collagen; COX—cyclooxygenase; CTNNB1—catenin beta-1 gene; CXCR—chemokine receptor; DISC1—DISC1 scaffold protein; ECM—extracellular matrix; ERK—extracellular signal-regulated kinases; ERα—estrogen receptor α; FGF14—fibroblast growth factor 14; FoxO1—forkhead box protein O1; GAS1—growth arrest-specific protein 1; GDF-5—growth/differentiation factor 5; GluA—ionotropic gluta-mate receptors; GSK3B—glycogen synthase kinase-3 beta; has-miR—human microRNA; HDAC4—histone deacetylase 4; HOXD10—homeobox D10; IL—interleukin; iNOS—inducible NO synthase; IVDD—intervertebral disc degeneration; IVD—intervertebral disc; KDM3A—lysine demethylase 3A; LINC00917—long intergenic non-protein coding RNA 917; LPS—lipopolysaccharide; MAPK—membrane-activated protein kinase; MMP—matrix metalloproteinase; mRNA—matrix ribonucleic acid; mTOR—mammalian target of rapamycin; MT1DP—metallothionein 1D, pseudogene; ND—no data; NF-κB—nuclear factor kappa B; NP—nucleus pulposus; NRF2—nuclear factor erythroid 2-related factor 2; PI3K-Akt—signaling pathway whose central components are the enzymes phosphoinositide 3-kinase, AKT, and mTOR; PTEN—phosphatase and tensin Homolog; RHOC—Ras homolog gene family, member C; RUNX-2—Runt-related transcription factor 2; p65—transcription factor p65; SDF1—stromal cell-derived factor-1; SIRT1—sirtuin 1 histone deacetylase; SMAD—signal transducers and transcriptional modulators of several signaling pathways; SOX-9—SRY-box transcription factor 9; STAT—activator of transcription; TARF6—TNF receptor-associated factor 6; TGF-β—transforming growth factor beta; TIMP4—metalloproteinase inhibitor 4; TLR—toll-like receptor; TNF—tumor necrosis factor; Wnt/β—Wnt signaling pathway.

## 4. Discussion

### 4.1. Perspectives of Using microRNAs as Epigenetic Biomarkers of Chronic Back Pain

**Figure 2:**
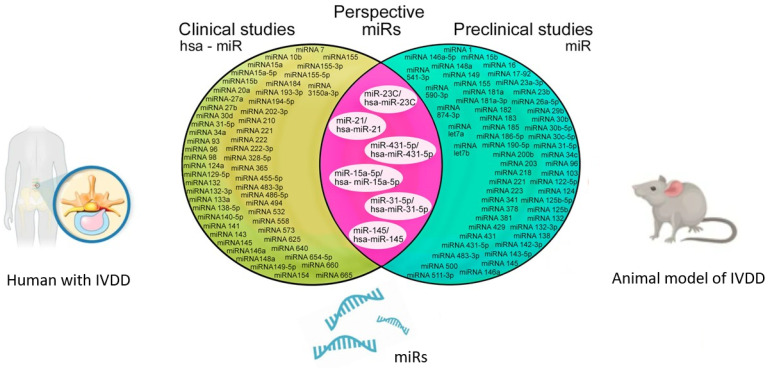
Perspective microRNAs as epigenetic biomarkers of chronic back pain in the patient with intervertebral disc degeneration. Note: Yellow circle—human microRNAs (miRs), a significant change in the expression of which was found in clinical studies involving patients with IVDD (in the blood, IVD tissues, spinal nerves, etc.); Green circle—animal miRs, a significant change in the expression of which was found in preclinical studies on various animal models (IVD tissues, spinal nerves, etc.); Pink—miRNAs with reproducible clinical evidence (these miRs are the most perspective for further bridge studies as epigenetic biomarkers of CBP in a patient with IVDD).

This review summarizes the results of preclinical and clinical studies on the role of local and circulating miRs in the formation and maintenance of the local and systemic inflammatory response, which is one of the main mechanisms for the development of CBP in patients with IVDD (Figure 2).

**Figure 3:**
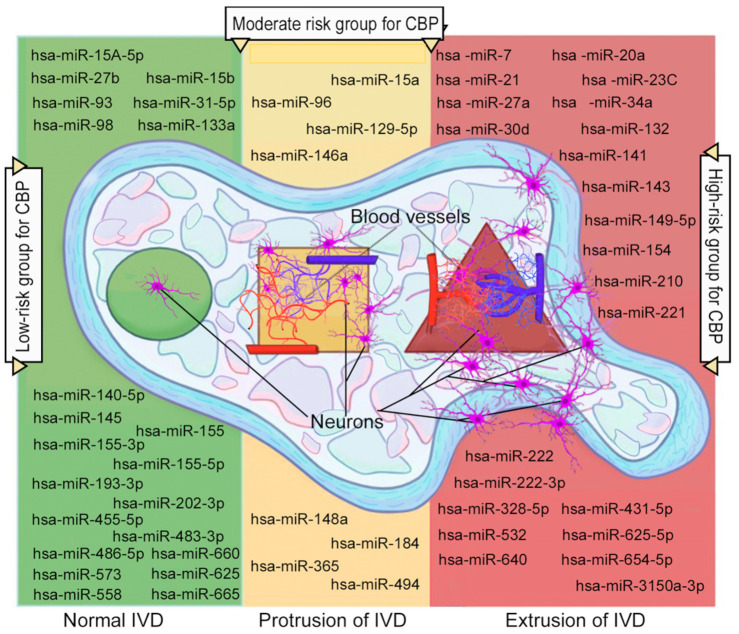
Risk groups of developing a systemic inflammatory response and chronic back pain syndrome in patients with intervertebral disk degeneration. Note: Green circle—low risk of development and progression of CBP (patients with IVDD without damage to the structures of the bone plate and protrusion of NP through AF); Yellow square—moderate risk of development and progression of CBP (patients with IVDD with the formation of tears of AF, protrusion of NP, and cell hyperproliferation and suppression of natural processes of programmed cell death, the onset of the formation of neo-angiogenesis and neo-neurogenesis); Red triangle—high risk of development and progression of CBP (patients with IVDD with damage to the structures of the endplate and AF, the formation of NP extrusion, and the formation of pathological proliferation of vessels and nerves). Abbreviations: AF—annulus fibrosis; NP—nucleus pulposus; CBP—chronic back pain; IVD—intervertebral disk; IVDD—intervertebral disk degeneration; miR—microRNA; hsa-miR—Homo Sapiens microRNA.

Next, miRs that had demonstrated significance in preclinical and clinical studies (miRs with reproducible clinical evidence) were selected (Figure 3). Patients with IVDD who were found to have elevated expression levels of predictive miRs that promoted the progression of the local and/or systemic inflammatory response were classified as having a high risk of developing CBP in IVDD. Patients with altered miR signature whose role is controversial were classified as having a moderate risk. Patients with elevated levels of predictive miRs that reduce the severity of the systemic inflammatory response were classified as having a low risk of developing CBP in IVDD (Figure 3).

**Figure 4:**
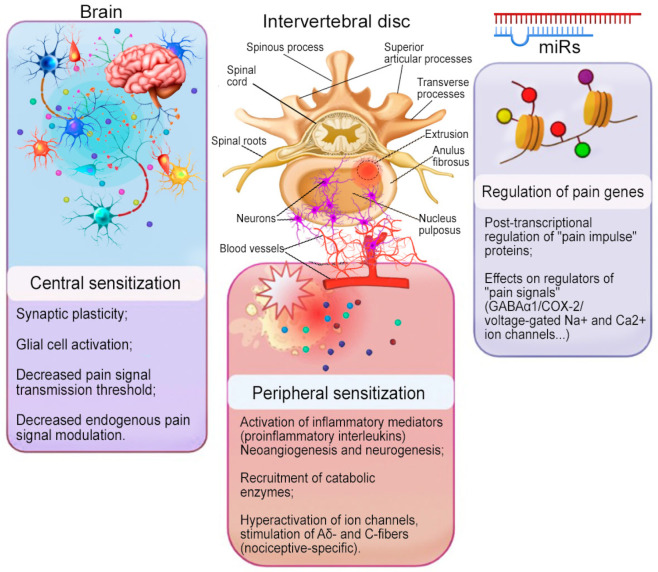
Mechanisms of development of chronic back pain syndrome via microRNA expression in patients with intervertebral disk degeneration. Note: Purple box—main mechanisms of central sensitization of discogenic back pain in patients with IVDD; Red box—main mechanisms of peripheral sensitization of discogenic back pain in patients with IVDD; Gray box—main mechanisms associated with microRNA-mediated regulation of discogenic back pain in patients with IVDD. Abbreviations: miR—microRNA; GABAα1—α1 subunit of gamma-aminobutyric acid receptor; COX—cyclooxygenase type 2.

Based on a summary of the results of our previous studies of the last decade [7,139,140], we identified the main mechanisms of CBP formation in IVDD, as well as the main proinflammatory and anti-inflammatory effects of the studied miRs (Figure 4).

As shown in Figure 2 (red triangle), the most perspective epigenetic biomarkers of high risk of CBP development and progression in patients with IVDD are the following (in experimental animals and humans, respectively): miR-15a-5p/hsa-miR-15a-5p, miR-21/hsa-miR-21, miR-23c/hsa-miR-23c, miR-31-5p/hsa-miR-31-5p, miR-132/hsa-miR-132, miR-145/hsa-miR-145, miR-146a/hsa-miR-146a, miR-148a/hsa-miR-148a, miR-155/hsa-miR-155, miR-221/hsa-miR-221, miR-431-5p/hsa-miR-431-5p, and miR-483-5p/hsa-miR-483-5p. However, it should be recognized that the assessment of the role of this miR signature should still be cautious. This is due to the heterogeneity of the designs of the studies we analyzed and the small sample sizes.

Clinical and preclinical studies have confirmed their similar effects as both predictive (miR-21/hsa-miR-21; miR-23c/hsa-miR-23c; and miR-431-5p/hsa-miR-431-5p) [42,78,86] and protective (miR-15a-5p/hsa-miR-15a-5p; miR-31-5p/hsa-miR-31-5p; and miR-145/hsa-miR-145) [58,60,61,90] epigenetic biomarkers for CBP (Figure 2, Yellow square). However, the role of some miRs (miR-132/hsa-miR-132; miR-146a/hsa-miR-146a; miR-148a/hsa-miR-148a; miR-155/hsa-miR-155; miR-221/hsa-miR-221; and miR-483-5p/hsa-miR-483-5p) [31,33,39,45,48,80,107,117,123] is ambiguous and it needs further study. The diagnostic value of these biomarkers is contradictory in preclinical (experimental) and clinical studies.

In the last decade, the focus of epigenetic research on IVDD has shifted from the study for miRs associated with the development and adverse course of this disease to an in-depth study of the therapeutic potential of miRs [8,141]: (1) unlike traditional symptomatic relief, miR therapies directly target the molecular drivers of a degenerating IVDD, such as NP cell apoptosis, extracellular matrix degradation, and inflammation; (2) the development of miR-based nanopreparation delivery systems into avascular degenerating IVD will allow achieving high biocompatibility and precise, prolonged miR release directly into the target NP and AF tissue); (3) the development of adaptive delivery systems focuses on stimulus-responsive materials that release therapeutic miRs into target tissues in response to the specific “hard” environment of a degenerating IVD (for example, changes in pH, reactive oxygen species, or specific enzymes). However, the therapeutic use of miRs in IVDD is still difficult, and the question of dosage in therapy using next-generation drugs based on miRs remains open. Also, the researchers realize that further research is needed to prevent adverse drug reactions during miR therapy.

We hope that future research will allow us not only to conduct large-scale bridge studies of the miR signature in IVDD and begin using it as a new diagnostic technology in real clinical practice, but also to develop new therapeutic strategies based on miR drugs.

### 4.2. Limitations of Using microRNAs as Epigenetic Biomarkers of Chronic Back Pain

The analyzed studies had variable designs and participant age characteristics. Most clinical studies assessed miR expression levels in tissue samples from degenerating and healthy IVDs obtained intraoperatively. Only a few publications have examined the role of circulating miRs in the blood (plasma, serum, and macrophages) in animal models of IVDD-specific CBP and in patients with IVDD. Several studies were heterogeneous and not age-matched, including children, adolescents, and adults. It should be recognized that there is a limited amount of information based on age and gender in the studies we analyzed, although these factors may affect miR expression and IVDD progression. The role of some miRs is controversial, as they have demonstrated both proinflammatory and anti-inflammatory effects or modulated the expression of CBP in patients with IVDD. These epigenetic biomarkers include the following: miR-34c, miR-132, miR-183, miR-221, miR-200b, miR-429, hsa-miR-15a, hsa-miR-96, hsa-miR-129-5p, hsa-miR-146a, hsa-miR-148a, hsa-miR-184, hsa-miR-365, and hsa-miR-494 [29,31,34,36,41,44,48,54,58,73,83,96,100,101,102,105,111,117]. Adding information about the stage of the disease to Table 2 would improve our review, since miR expression also depends on the stage of the degenerative process and the chronicity of back pain. However, such information was missing from most of the publications that we analyzed.

Also, within the framework of a single scoping review, it is difficult to include the entire volume of already published and currently published works devoted to such a broad problem as epigenetic modifications of the processes of repair, regeneration, degeneration of IVD, the development of nociceptive, neuropathic components of pain syndrome, and the risks and stages of development of herniated disc protrusions at the lumbosacral and other levels [142,143,144,145,146,147,148,149,150,151,152,153,154,155].

Despite the perspective results of the studies analyzed, there are limitations to the use of miRs as predictive epigenetic biomarkers for CBP in patients with IVDD in real-world clinical practice, as miRs signature in relation to the mechanisms of CBP formation and progression in IVDD is still poorly understood. Future studies on the role of miRs may provide further direction for the development of personalized approaches to both CBP treatment and other areas of personalized neuroscience [156].

There are numerous limitations in using miRs as epigenetic biomarkers of CBP in patients with IVDD in real clinical practice: (1) it is unknown why and to what extent miRs are selectively exported, including during the systemic inflammatory response [157]; (2) numerous quality issues in previous studies related to sample collection, hemolysis, and low yields of miR extraction; (3) the influence of various hormonal and metabolic factors on plasma miR expression levels [141]; and (4) the influence of comorbid diseases (e.g., diabetes mellitus, obesity, etc.) on miR signature for CBP in patients with IVDD [4].

## 5. Conclusions

In this review, it was shown that miRs may play an important role in the epigenetic regulation of inflammatory and degenerative processes in IVDD, acting as biomarkers for assessing the risk and predicting the severity of CBP. The most studied predictive miRs are hsa-miR-15a-5p, hsa-miR-21, hsa-miR-23c, hsa-miR-31-5p, hsa-miR-145, and hsa-miR-431-5p, which demonstrated a significant effect on inflammatory pathways and the formation of CBP in preclinical experiments (using other animal models) and clinical trials involving patients with IVDD. In the future, previously studied miRs with specificity and sensitivity can be considered as epigenetic biomarkers (predictors) of the risk of developing and severity of CBP in patients with IVDD. However, limited knowledge about the role of miRs signatures must be considered due to the variability of miR expression and the influence of comorbid diseases. Possible, further miR research may open new avenues for targeted therapy of CBP in patients with IVDD, as well as personalized assessment of risk factors for severe IVDD based on the presence of these epigenetic biomarkers.

## Data Availability

No new data were created or analyzed in this study. Data sharing is not applicable to this article.

## Supplementary Materials

The following supporting information can be downloaded at https://www.mdpi.com/article/10.3390/ijms27031167/s1.

## Abbreviations

AF	annulus fibrosis
BDNF	brain-derived neurotrophic factor
CBP	chronic back pain
CGRP	calcitonin gene-related peptide
COX-2	cyclooxygenase 2
GABA-α1	gamma-aminobutyric acid α1
GM-CSF	granulocyte–macrophage colony-stimulating factor
IL1-β	interleukin 1 beta
IVD	intervertebral disc
IVDD	intervertebral disc degeneration
microRNA	small non-coding ribonucleic acid
NGF	nerve growth factor
NP	nucleus pulposus
PGE-2	prostaglandin E2

## References

[1] Kirnaz S., Capadona C., Wong T., Goldberg J.L., Medary B., Sommer F., McGrath L.B., Härtl R. (2022). Fundamentals of intervertebral disc degeneration. World Neurosurg..

[2] Kang L., Zhang H., Jia C., Zhang R., Shen C. (2023). Epigenetic modifications of inflammation in intervertebral disc degeneration. Ageing Res. Rev..

[3] Silwal P., Nguyen-Thai A.M., Mohammad H.A., Wang Y., Robbins P.D., Lee J.Y., Vo N.V. (2023). Cellular senescence in intervertebral disc aging and degeneration: Molecular mechanisms and potential therapeutic opportunities. Biomolecules.

[4] Cazzanelli P., Wuertz-Kozak K. (2020). MicroRNAs in intervertebral disc degeneration, apoptosis, inflammation, and mechanobiology. Int. J. Mol. Sci..

[5] Chang C., Xu L., Zhang R., Jin Y., Jiang P., Wei K., Xu L., Shi Y., Zhao J., Xiong M. (2022). MicroRNA-mediated epigenetic regulation of rheumatoid arthritis susceptibility and pathogenesis. Front. Immunol..

[6] Shnayder N.A., Ashkhotov A.V., Trefilova V.V., Nurgaliev Z.A., Novitsky M.A., Petrova M.M., Narodova E.A., Al-Zamil M., Chumakova G.A., Garganeeva N.P. (2023). Molecular basic of pharmacotherapy of cytokine imbalance as a component of intervertebral disc degeneration treatment. Int. J. Mol. Sci..

[7] Shnayder N.A., Ashhotov A.V., Trefilova V.V., Nurgaliev Z.A., Novitsky M.A., Vaiman E.E., Petrova M.M., Nasyrova R.F. (2023). Cytokine imbalance as a biomarker of intervertebral disk degeneration. Int. J. Mol. Sci..

[8] Shnayder N.A., Ashhotov A.V., Trefilova V.V., Novitsky M.A., Medvedev G.V., Petrova M.M., Narodova E.A., Kaskaeva D.S., Chumakova G.A., Garganeeva N.P. (2023). High-tech methods of cytokine imbalance correction in intervertebral disc degeneration. Int. J. Mol. Sci..

[9] Henry N., Clouet J., Le Bideau J., Le Visage C., Guicheux J. (2018). Innovative strategies for intervertebral disc regenerative medicine: From cell therapies to multiscale delivery systems. Biotechnol. Adv..

[10] Clouet J., Fusellier M., Camus A., Le Visage C., Guicheux J. (2019). Intervertebral disc regeneration: From cell therapy to the development of novel bioinspired endogenous repair strategies. Adv. Drug Deliv. Rev..

[11] Li W., Gong Y., Liu J., Guo Y., Tang H., Qin S., Zhao Y., Wang S., Xu Z., Chen B. (2021). Peripheral and Central Pathological Mechanisms of Chronic Low Back Pain: A Narrative Review. J. Pain Res..

[12] De Geer C.M. (2018). Cytokine involvement in biological inflammation related to degenerative disorders of the intervertebral disk: A narrative review. J. Chiropr. Med..

[13] Fujii K., Yamazaki M., Kang J.D., Risbud M.V., Cho S.K., Qureshi S.A., Hecht A.C., Iatridis J.C. (2019). Discogenic Back Pain: Literature Review of Definition, Diagnosis, and Treatment. JBMR Plus.

[14] Smith P.A. (2024). BDNF in neuropathic pain; the culprit that cannot be apprehended. Neuroscience.

[15] Michell-Robinson M.A., Touil H., Healy L.M., Owen D.R., Durafourt B.A., Bar-Or A., Antel J.P., Moore C.S. (2015). Roles of microglia in brain development, tissue maintenance and repair. Brain.

[16] Sengupta J.N., Pochiraju S., Kannampalli P., Bruckert M., Addya S., Yadav P., Miranda A., Shaker R., Banerjee B. (2013). MicroRNA-mediated GABA Aα-1 receptor subunit down-regulation in adult spinal cord following neonatal cystitis-induced chronic visceral pain in rats. Pain.

[17] Park S.J., Cheon E.J., Kim H.A. (2013). MicroRNA-558 regulates the expression of cyclooxygenase-2 and IL-1β-induced catabolic effects in human articular chondrocytes. Osteoarthr. Cartil..

[18] Bourinet E., Altier C., Hildebrand M.E., Trang T., Salter M.W., Zamponi G.W. (2014). Calcium-permeable ion channels in pain signaling. Physiol. Rev..

[19] Ni J., Gao Y., Gong S., Guo S., Hisamitsu T., Jiang X. (2013). Regulation of μ-opioid type 1 receptors by microRNA134 in dorsal root ganglion neurons following peripheral inflammation. Eur. J. Pain.

[20] Im H.I., Kenny P.J. (2012). MicroRNAs in neuronal function and dysfunction. Trends Neurosci..

[21] Tricco A.C., Lillie E., Zarin W., O’Brien K.K., Colquhoun H., Levac D., Moher D., Peters M.D.J., Horsley T., Weeks L. (2018). PRISMA Extension for Scoping Reviews (PRISMAScR): Checklist and Explanation. Ann. Intern. Med..

[22] Zhou X., Chen L., Grad S., Alini M., Pan H., Yang D., Zhen W., Li Z., Huang S., Peng S. (2017). The roles and perspectives of microRNAs as biomarkers for intervertebral disc degeneration. J. Tissue Eng. Regen. Med..

[23] Xie X., Ma L., Xi K., Zhang W., Fan D. (2017). MicroRNA-183 suppresses neuropathic pain and expression of AMPA receptors by targeting mTOR/VEGF signaling pathway. Cell. Physiol. Biochem..

[24] Neumann E., Hermanns H., Barthel F., Werdehausen R., Brandenburger T. (2015). Expression changes of microRNA-1 and its targets Connexin 43 and brain-derived neurotrophic factor in the peripheral nervous system of chronic neuropathic rats. Mol. Pain.

[25] Hori N., Narita M., Yamashita A., Horiuchi H., Hamada Y., Kondo T., Watanabe M., Igarashi K., Kawata M., Shibasaki M. (2016). Changes in the expression of IL-6-Mediated MicroRNAs in the dorsal root ganglion under neuropathic pain in mice. Synapse.

[26] Pan Z., Shan Q., Gu P., Wang X.M., Tai L.W., Sun M., Luo X., Sun L., Cheung C.W. (2018). miRNA-23a/CXCR4 regulates neuropathic pain via directly targeting TXNIP/NLRP3 inflammasome axis. J. Neuroinflamm..

[27] Zhang Y., Su Z., Liu H.L., Li L., Wei M., Ge D.J., Zhang Z.J. (2018). Effects of miR-26a-5p on neuropathic pain development by targeting MAPK6 in in CCI rat models. Biomed. Pharmacother..

[28] Tramullas M., Francés R., de la Fuente R., Velategui S., Carcelén M., García R., Llorca J., Hurlé M.A. (2018). MicroRNA-30c-5p modulates neuropathic pain in rodents. Sci. Transl. Med..

[29] Xu L., Wang Q., Jiang W., Yu S., Zhang S. (2019). MiR-34c ameliorates neuropathic pain by targeting NLRP3 in a mouse model of chronic constriction injury. Neuroscience.

[30] Bali K.K., Hackenberg M., Lubin A., Kuner R., Devor M. (2014). Sources of individual variability: miRNAs that predispose to neuropathic pain identified using genome-wide sequencing. Mol. Pain.

[31] Liu W., Xia P., Feng J., Kang L., Huang M., Wang K., Song Y., Li S., Wu X., Yang S. (2017). MicroRNA-132 upregulation promotes matrix degradation in intervertebral disc degeneration. Exp. Cell Res..

[32] Li X., Wang S., Yang X., Chu H. (2021). miR1423p targets AC9 to regulate sciatic nerve injuryinduced neuropathic pain by regulating the cAMP/AMPK signalling pathway. Int. J. Mol. Med..

[33] Sun J., Hong J., Sun S., Wang X., Peng Y., Zhou J., Huang Y., Li S., Chen W., Li C. (2018). Transcription factor 7-like 2 controls matrix degradation through nuclear factor κB signaling and is repressed by microRNA-155 in nucleus pulposus cells. Biomed. Pharmacother..

[34] Huang L., Wang L. (2020). Upregulation of miR-183 represses neuropathic pain through inhibiton of MAP3K4 in CCI rat models. J. Cell. Physiol..

[35] Li L., Zhao G. (2016). Downregulation of microRNA-218 relieves neuropathic pain by regulating suppressor of cytokine signaling 3. Int. J. Mol. Med..

[36] Wu X., Wang X., Yin Y., Zhu L., Zhang F., Yang J. (2021). Investigation of the role of miR-221 in diabetic peripheral neuropathy and related molecular mechanisms. Adv. Clin. Exp. Med..

[37] Li H., Shen L., Ma C., Huang Y. (2013). Differential expression of miRNAs in the nervous system of a rat model of bilateral sciatic nerve chronic constriction injury. Int. J. Mol. Med..

[38] Sheng X., Guo Q., Yu J., Xu Y. (2018). Experimental research on the effect of microRNA-21 inhibitor on a rat model of intervertebral disc degeneration. Exp. Ther. Med..

[39] Yang S., Li L., Zhu L., Zhang C., Li Z., Guo Y., Nie Y., Luo Z. (2019). Bu-Shen-Huo-Xue-Fang modulates nucleus pulposus cell proliferation and extracellular matrix remodeling in intervertebral disk degeneration through miR-483 regulation of Wnt pathway. J. Cell. Biochem..

[40] Su S., Zhao L., Xie W., Yi D., He S., Chen D., Huang J. (2019). Serum miRNAs are potential biomarkers for the detection of disc degeneration, among which miR-26a-5p suppresses Smad1 to regulate disc homeostasis. J. Cell. Mol. Med..

[41] Zhang S., Song S., Cui W., Liu X., Sun Z. (2022). Mechanism of long Noncoding RNA HOTAIR in nucleus pulposus cell autophagy and apoptosis in intervertebral disc degeneration. Evid. Based Complement. Altern. Med..

[42] Wang H., Zhu Y., Cao L., Guo Z., Sun K., Qiu W., Fan H. (2021). circARL15 plays a critical role in intervertebral disc degeneration by modulating miR-431-5p/DISC1. Front. Genet..

[43] Kynast K.L., Russe O.Q., Möser C.V., Geisslinger G., Niederberger E. (2013). Modulation of central nervous system-specific microRNA-124a alters the inflammatory response in the formalin test in mice. Pain.

[44] Arai M., Genda Y., Ishikawa M., Shunsuke T., Okabe T., Sakamoto A. (2013). The miRNA and mRNA changes in rat hippocampi after chronic constriction injury. Pain Med..

[45] Zhang R., Huang M., Cao Z., Qi J., Qiu Z., Chiang L.Y. (2015). MeCP2 plays an analgesic role in pain transmission through regulating CREB/miR-132 pathway. Mol. Pain.

[46] Jin Y., Xu L., Xu Y. (2021). Effect of intrathecal injection of miRNA-138 on neuropathic pain in rats undergoing partial sciatic nerve ligation and its underlying mechanism. Ann. Palliat. Med..

[47] Kameshima S., Okada M., Ikeda S., Watanabe Y., Yamawaki H. (2016). Coordination of changes in expression and phosphorylation of eukaryotic elongation factor 2 (eEF2) and eEF2 kinase in hypertrophied cardiomyocytes. Biochem. Biophys. Rep..

[48] Li X., Kroin J.S., Kc R., Gibson G., Chen D., Corbett G.T., Pahan K., Fayyaz S., Kim J.S., van Wijnen A.J. (2013). Altered spinal microRNA-146a and the microRNA-183 cluster contribute to osteoarthritic pain in knee joints. J. Bone Min. Res..

[49] Wang Z., Liu F., Wei M., Qiu Y., Ma C., Shen L., Huang Y. (2018). Chronic constriction injury-induced microRNA-146a-5p alleviates neuropathic pain through suppression of IRAK1/TRAF6 signaling pathway. J. Neuroinflamm..

[50] Qin C., Lv Y., Zhao H., Yang B., Zhang P. (2019). MicroRNA-149 Suppresses Inflammation in Nucleus Pulposus Cells of Intervertebral Discs by Regulating MyD88. Med. Sci. Monit..

[51] Yun Z., Wang Y., Feng W., Zang J., Zhang D., Gao Y. (2020). Overexpression of microRNA-185 alleviates intervertebral disc degeneration through inactivation of the Wnt/beta-catenin signaling pathway and downregulation of Galectin-3. Mol. Pain.

[52] Jiang B.C., Cao D.L., Zhang X., Zhang Z.J., He L.N., Li C.H., Zhang W.W., Wu X.B., Berta T., Ji R.R. (2016). CXCL13 drives spinal astrocyte activation and neuropathic pain via CXCR5. J. Clin. Investig..

[53] Yang D., Yang Q., Wei X., Liu Y., Ma D., Li J., Wan Y., Luo Y. (2017). The role of miR-190a-5p contributes to diabetic neuropathic pain via targeting SLC17A6. J. Pain Res..

[54] Yan X.T., Zhao Y., Cheng X.L., He X.H., Wang Y., Zheng W.Z., Chen H., Wang Y.L. (2018). Inhibition of miR-200b/miR-429 contributes to neuropathic pain development through targeting zinc finger E box binding protein-1. J. Cell. Physiol..

[55] Zhu J., Yang J., Xu J. (2021). miR-223 inhibits the polarization and recruitment of macrophages via NLRP3/IL-1β pathway to meliorate neuropathic pain. Pain Res. Manag..

[56] Gao P., Zeng X., Zhang L., Wang L., Shen L.L., Hou Y.Y., Zhou F., Zhang X. (2021). Overexpression of miR-378 Alleviates Chronic Sciatic Nerve Injury by Targeting EZH2. Neurochem. Res..

[57] Zhan L.Y., Lei S.Q., Zhang B.H., Li W.L., Wang H.X., Zhao B., Cui S.S., Ding H., Huang Q.M. (2018). Overexpression of miR-381 relieves neuropathic pain development via targeting HMGB1 and CXCR4. Biomed. Pharmacother..

[58] Jiang C., Chen Z., Wang X., Zhang Y., Guo X., Xu Z., Yang H., Hao D. (2022). The potential mechanisms and application prospects of non-coding RNAs in intervertebral disc degeneration. Front. Endocrinol..

[59] Du K., He X., Deng J. (2018). MicroRNA-16 inhibits the lipopolysaccharide-induced inflammatory response in nucleus pulposus cells of the intervertebral disc by targeting TAB3. Arch. Med. Sci..

[60] Zhou Y., Deng M., Su J., Zhang W., Liu D., Wang Z. (2021). The role of miR-31-5p in the development of intervertebral disc degeneration and its therapeutic potential. Front. Cell Dev. Biol..

[61] Xie L., Chen Z., Liu M., Huang W., Zou F., Ma X., Tao J., Guo J., Xia X., Lyu F. (2020). MSC-Derived Exosomes Protect Vertebral Endplate Chondrocytes against Apoptosis and Calcification via the miR-31-5p/ATF6 Axis. Mol. Ther. Nucleic Acids.

[62] Wang X., Wang Q., Li G., Xu H., Liu B., Yuan B., Zhou Y., Li Y. (2024). Identifying the protective effects of miR-874-3p/ATF3 axis in intervertebral disc degeneration by single-cell RNA sequencing and validation. J. Cell. Mol. Med..

[63] Ito N., Sakai A., Miyake N., Maruyama M., Iwasaki H., Miyake K., Okada T., Sakamoto A., Suzuki H. (2017). miR-15b mediates oxaliplatin-induced chronic neuropathic pain through BACE1 down-regulation. Br. J. Pharmacol..

[64] Sakai A., Saitow F., Maruyama M., Miyake N., Miyake K., Shimada T., Okada T., Suzuki H. (2017). MicroRNA cluster miR-17-92 regulates multiple functionally related voltage-gated potassium channels in chronic neuropathic pain. Nat. Commun..

[65] Im Y.B., Jee M.K., Choi J.I., Cho H.T., Kwon O.H., Kang S.K. (2012). Molecular targeting of NOX4 for neuropathic pain after traumatic injury of the spinal cord. Cell Death Dis..

[66] Su S., Shao J., Zhao Q., Ren X., Cai W., Li L., Bai Q., Chen X., Xu B., Wang J. (2017). MiR-30b Attenuates Neuropathic Pain by Regulating Voltage-Gated Sodium Channel Nav1.3 in Rats. Front. Mol. Neurosci..

[67] Li L., Shao J., Wang J., Liu Y., Zhang Y., Zhang M., Zhang J., Ren X., Su S., Li Y. (2019). MiR-30b-5p attenuates oxaliplatin-induced peripheral neuropathic pain through the voltage-gated sodium channel Nav1.6 in rats. Neuropharmacology.

[68] Chen H.P., Zhou W., Kang L.M., Yan H., Zhang L., Xu B.H., Cai W.H. (2014). Intrathecal miR-96 inhibits Nav1.3 expression and alleviates neuropathic pain in rat following chronic construction injury. Neurochem. Res..

[69] Favereaux A., Thoumine O., Bouali-Benazzouz R., Roques V., Papon M.A., Salam S.A., Drutel G., Léger C., Calas A., Nagy F. (2011). Bidirectional integrative regulation of Cav1.2 calcium channel by microRNA miR-103: Role in pain. EMBO J..

[70] Wan L., Su Z., Li F., Gao P., Zhang X. (2021). MiR-122-5p suppresses neuropathic pain development by targeting PDK4. Neurochem. Res..

[71] Leinders M., Uceyler N., Pritchard R.A., Sommer C., Sorkin L.S. (2016). Increased miR-132-3p expression is associated with chronic neuropathic pain. Exp. Neurol..

[72] Aldrich B.T., Frakes E.P., Kasuya J., Hammond D.L., Kitamoto T. (2009). Changes in expression of sensory organ-specific microRNAs in rat dorsal root ganglia in association with mechanical hypersensitivity induced by spinal nerve ligation. Neuroscience.

[73] Imai S., Saeki M., Yanase M., Horiuchi H., Abe M., Narita M., Kuzumaki N., Suzuki T., Narita M. (2011). Change in microRNAs associated with neuronal adaptive responses in the nucleus accumbens under neuropathic pain. J. Neurosci..

[74] Huang Z.Z., Wei J.Y., Ou-Yang H.D., Li D., Xu T., Wu S.L., Zhang X.L., Liu C.C., Ma C., Xin W.J. (2016). mir-500-Mediated GAD67 Downregulation Contributes to Neuropathic Pain. J. Neurosci..

[75] Sakai A., Saitow F., Miyake N., Miyake K., Shimada T., Suzuki H. (2013). miR-7a alleviates the maintenance of neuropathic pain through regulation of neuronal excitability. Brain.

[76] Park C.K., Xu Z.Z., Berta T., Han Q., Chen G., Liu X.J., Ji R.R. (2014). Extracellular microRNAs activate nociceptor neurons to elicit pain via TLR7 and TRPA1. Neuron.

[77] Murphy C.P., Singewald N. (2019). Role of MicroRNAs in anxiety and anxiety-related disorders. Curr. Top. Behav. Neurosci..

[78] Sun Y., Shi X., Peng X., Li Y., Ma H., Li D., Cao X. (2020). MicroRNA-181a exerts anti-inflammatory effects via inhibition of the ERK pathway in mice with intervertebral disc degeneration. J. Cell. Physiol..

[79] Zhang Z.J., Guo J.S., Li S.S., Wu X.B., Cao D.L., Jiang B.C., Jing P.B., Bai X.Q., Li C.H., Wu Z.H. (2018). TLR8 and its endogenous ligand miR-21 contribute to neuropathic pain in murine DRG. J. Exp. Med..

[80] Willemen H.L., Huo X.J., Mao-Ying Q.L., Zijlstra J., Heijnen C.J., Kavelaars A. (2012). MicroRNA-124 as a novel treatment for persistent hyperalgesia. J. Neuroinflamm..

[81] Li X., Gibson G., Kim J.S., Kroin J., Xu S., van Wijnen A.J., Im H.J. (2011). MicroRNA-146a is linked to pain-related pathophysiology of osteoarthritis. Gene.

[82] Poh K.W., Yeo J.F., Ong W.Y. (2011). MicroRNA changes in the mouse prefrontal cortex after inflammatory pain. Eur. J. Pain.

[83] Xia L., Zhang Y., Dong T. (2016). Inhibition of microRNA-221 alleviates neuropathic pain through targeting suppressor of cytokine signaling 1. J. Mol. Neurosci..

[84] Shen L., Xiao Y., Wu Q., Liu L., Zhang C., Pan X. (2019). TLR4/NF-κB Axis Signaling Pathway-Dependent Up-Regulation of miR-625-5p Contributes to Human Intervertebral Disc Degeneration by Targeting COL1A1. Am. J. Transl. Res..

[85] Xiao L., Xu S., Xu Y., Liu C., Yang B., Wang J., Xu H. (2018). TGF-beta/SMAD signaling inhibits intermittent cyclic mechanical tension-induced degeneration of endplate chondrocytes by regulating the miR-455-5p/RUNX2 axis. J. Cell. Biochem..

[86] Wang W.J., Yang W., Ouyang Z.H., Xue J.B., Li X.L., Zhang J., He W.S., Chen W.K., Yan Y.G., Wang C. (2018). MiR-21 promotes ECM degradation through inhibiting autophagy via the PTEN/akt/mTOR signaling pathway in human degenerated NP cells. Biomed. Pharmacother..

[87] Chen H., Wang J., Hu B., Wu X., Chen Y., Li R., Yuan W. (2015). MiR-34a pro-motes Fas-mediated cartilage end-plate chondro-cyte apoptosis by targeting Bcl-2. Mol. Cell. Biochem..

[88] Dong W., Liu J., Lv Y., Wang F., Liu T., Sun S., Liao B., Shu Z., Qian J. (2019). miR-640 aggravates intervertebral disc degeneration via NF-kappaB and WNT signalling pathway. Cell Prolif..

[89] Li H.R., Cui Q., Dong Z.Y., Zhang J.H., Li H.Q., Zhao L. (2016). Downregulation of miR-27b is Involved in Loss of Type II Collagen by Directly Targeting Matrix Metalloproteinase 13 (MMP13) in Human Intervertebral Disc Degeneration. Spine.

[90] Zhou J., Sun J., Markova D.Z., Li S., Kepler C.K., Hong J., Huang Y., Chen W., Xu K., Wei F. (2019). MicroRNA-145 overexpression attenuates apoptosis and increases matrix synthesis in nucleus pulposus cells. Life Sci..

[91] Zhang H.J., Ma X.H., Xie S.L., Qin S.L., Liu C.Z., Zhang Z.G. (2020). Knockdown of miR-660 protects nucleus pulposus cells from TNF-a-induced apoptosis by targeting serum amyloid A1. J. Orthop. Surg. Res..

[92] Ji M.L., Lu J., Shi P.L., Zhang X.J., Wang S.Z., Chang Q., Chen H., Wang C. (2016). Dysregulated miR-98 contributes to extracellular matrix degradation by targeting IL-6/STAT3 signaling pathway in human intervertebral disc degeneration. J. Bone Miner. Res..

[93] Liu M.H., Sun C., Yao Y., Fan X., Liu H., Cui Y.H., Bian X.W., Huang B., Zhou Y. (2016). Matrix stiffness promotes cartilage endplate chondrocyte calcification in disc degeneration via miR-20a targeting ANKH expression. Sci. Rep..

[94] Ji M.L., Jiang H., Zhang X.J., Shi P.L., Li C., Wu H., Wu X.T., Wang Y.T., Wang C., Lu J. (2018). Preclinical development of a microRNA-based therapy for intervertebral disc degeneration. Nat. Commun..

[95] Zhao K., Zhang Y., Kang L., Song Y., Wang K., Li S., Wu X., Hua W., Shao Z., Yang S. (2017). Epigenetic silencing of miRNA-143 regulates apoptosis by targeting BCL2 in human intervertebral disc degeneration. Gene.

[96] Kang L., Yang C., Song Y., Zhao K., Liu W., Hua W., Wang K., Tu J., Li S., Yin H. (2017). MicroRNA-494 promotes apoptosis and extracellular matrix degradation in degenerative human nucleus pulposus cells. Oncotarget.

[97] Cai P., Yang T., Jiang X., Zheng M., Xu G., Xia J. (2017). Role of miR-15a in intervertebral disc degeneration through targeting MAP3K9. Biomed. Pharmacother..

[98] Cao Z., Chen L. (2017). Inhibition of miR-27a suppresses the inflammatory response via the p38/MAPK pathway in intervertebral disc cells. Exp. Ther. Med..

[99] Lv J., Li S., Wan T., Yang Y., Cheng Y., Xue R. (2018). Inhibition of microRNA-30d attenuates the apoptosis and extracellular matrix degradation of degenerative human nucleus pulposus cells by up-regulating SOX9. Chem. Biol. Interact..

[100] Tao B., Yi J., Huang C., Xu W., Qin C., Chen L., Chen J., Gao Y., Wang R. (2017). microRNA-96 regulates the proliferation of nucleus pulposus cells by targeting ARID2/AKT signaling. Mol. Med. Rep..

[101] Yang W., Sun P. (2019). Downregulation of microRNA-129-5p increases the risk of intervertebral disc degeneration by promoting the apoptosis of nucleus pulposus cells via targeting BMP2. J. Cell. Biochem..

[102] Zhou T., Lin H., Cheng Z., Ji C., Zhang C., Tian J. (2017). Mechanism of microRNA-146a-mediated IL-6/STAT3 signaling in lumbar intervertebral disc degeneration. Exp. Ther. Med..

[103] Li T., Peng Y., Chen Y., Huang X., Li X., Zhang Z., Du J. (2022). Long intergenic non-coding RNA-00917 regulates the proliferation, inflammation, and pyroptosis of nucleus pulposus cells via targeting miR-149-5p/NOD-like receptor protein 1 axis. Bioengineered.

[104] Wang J., Liu X., Sun B., Du W., Zheng Y., Sun Y. (2019). Upregulated miR-154 promotes ECM degradation in intervertebral disc degeneration. J. Cell. Biochem..

[105] Li W., Wang P., Zhang Z., Wang W., Liu Y., Qi Q. (2017). MiR-184 regulates proliferation in nucleus pulposus cells by targeting GAS1. World Neurosurg..

[106] Wang C., Zhang Z.Z., Yang W., Ouyang Z.H., Xue J.B., Li X.L., Zhang J., Chen W.K., Yan Y.G., Wang W.J. (2017). MiR-210 facilitates ECM degradation by suppressing autophagy via silencing of ATG7 in human degenerated NP cells. Biomed. Pharmacother..

[107] Sheng B., Yuan Y., Liu X., Zhang Y., Liu H., Shen X., Liu B., Chang L. (2018). Protective effect of estrogen against intervertebral disc degeneration is attenuated by miR-221 through targeting estrogen receptor α. Acta Biochim. Biophys. Sin..

[108] Zhang Y., Yang J., Zhou X., Wang N., Li Z., Zhou Y., Feng J., Shen D., Zhao W. (2019). Knockdown of miR-222 inhibits inflammation and the apoptosis of LPS-stimulated human intervertebral disc nucleus pulposus cells. Int. J. Mol. Med..

[109] Liu J., Yu J., Jiang W., He M., Zhao J. (2019). Targeting of CDKN1B by miR-222-3p may contribute to the development of intervertebral disc degeneration. FEBS Open Bio.

[110] Yan J., Wu L.G., Zhang M., Fang T., Pan W., Zhao J.L., Zhou Q. (2022). miR-328-5p induces human intervertebral disc degeneration by targeting WWP2. Oxidative Med. Cell. Longev..

[111] Liao Z.W., Fan Z.W., Huang Y., Liang C.Y., Liu C., Huang S., Chen C.W. (2021). Long non-coding RNA MT1DP interacts with miR-365 and induces apoptosis of nucleus pulposus cells by repressing NRF-2-induced anti-oxidation in lumbar disc herniation. Ann. Transl. Med..

[112] Sun Z., Jian Y., Fu H., Li B. (2018). MiR-532 downregulation of the Wnt/β-catenin signaling via targeting Bcl-9 and induced human intervertebral disc nucleus pulposus cells apoptosis. J. Pharmacol. Sci..

[113] Wang S., Guo Y., Zhang X., Wang C. (2021). miR-654-5p inhibits autophagy by targeting ATG7 via mTOR signaling in intervertebral disc degeneration. Mol. Med. Rep..

[114] Zhang B., Guo W., Sun C., Duan H.Q., Yu B.B., Mu K., Guan Y.Y., Li Y., Liu S., Liu Y. (2018). Dysregulated miR-3150a-3p promotes lumbar intervertebral disc degeneration by targeting aggrecan. Cell. Physiol. Biochem..

[115] Jing W., Jiang W. (2015). MicroRNA-93 regulates collagen loss by targeting MMP3 in human nucleus pulposus cells. Cell Prolif..

[116] Xu C., Zhang H., Zhou W., Wu H., Shen X., Chen Y., Liao M., Liu Y., Yuan W. (2019). MicroRNA-10a, -210, and -563 as circulating biomarkers for ossification of the posterior longitudinal ligament. Spine J..

[117] Heyn J., Luchting B., Hinske L.C., Hubner M., Azad S.C., Kreth S. (2016). miR-124a and miR-155 enhance differentiation of regulatory T cells in patients with neuropathic pain. J. Neuroinflamm..

[118] Ji M.L., Zhang X.J., Shi P.L., Lu J., Wang S.Z., Chang Q., Chen H., Wang C. (2016). Downregulation of microRNA-193a-3p is involved in invertebral disc degeneration by targeting MMP14. J. Mol. Med..

[119] Zheng Q., Li X.X., Xiao L., Shao S., Jiang H., Zhang X.L., Sun L.Y., Xu H.G. (2019). MicroRNA-365 functions as a mechanosensitive microRNA to inhibit end plate chondrocyte degeneration by targeting histone deacetylase 4. Bone.

[120] Kang L., Yang C., Yin H., Zhao K., Liu W., Hua W., Wang K., Song Y., Tu J., Li S. (2017). MicroRNA-15b silencing inhibits IL-1β-induced extracellular matrix degradation by targeting SMAD3 in human nucleus pulposus cells. Biotechnol. Lett..

[121] Cui S., Zhang L. (2021). microRNA-129-5p shuttled by mesenchymal stem cell-derived extracellular vesicles alleviates intervertebral disc degeneration via blockade of LRG1-mediated p38 MAPK activation. J. Tissue Eng..

[122] Zhang Q., Weng Y., Jiang Y., Zhao S., Zhou D., Xu N. (2018). Overexpression of miR-140-5p inhibits lipopolysaccharide-induced human intervertebral disc inflammation and degeneration by downregulating toll-like receptor 4. Oncol. Rep..

[123] Lv F., Huang Y., Lv W., Yang L., Li F., Fan J., Sun J. (2017). MicroRNA-146a Ameliorates inflammation via TRAF6/NF-κB pathway in intervertebral disc cells. Med. Sci. Monit..

[124] Li G., Tang X., Chen H., Sun W., Yuan F. (2018). miR-148a inhibits pro-inflammatory cytokines released by intervertebral disc cells by regulating the p38/MAPK pathway. Exp. Ther. Med..

[125] Ye D., Dai L., Yao Y., Qin S., Xie H., Wang W., Liang W. (2016). miR-155 inhibits nucleus pulposus cells’ degeneration through targeting ERK 1/2. Dis. Markers.

[126] Zhou X., Li J., Teng J., Liu Y., Zhang D., Liu L., Zhang W. (2021). microRNA-155-3p attenuates intervertebral disc degeneration via inhibition of KDM3A and HIF1α. Inflamm. Res..

[127] Cazzanelli P., Lamoca M., Hasler J., Hausmann O.N., Mesfin A., Puvanesarajah V., Hitzl W., Wuertz-Kozak K. (2024). The role of miR-155-5p in inflammation and mechanical loading during intervertebral disc degeneration. Cell Commun. Signal..

[128] Shi C., Wu L., Lin W., Cai Y., Zhang Y., Hu B., Gao R., Im H.J., Yuan W., Ye X. (2019). MiR-202-3p regulates interleukin-1β-induced expression of matrix metalloproteinase 1 in human nucleus pulposus. Gene.

[129] Chai X., Si H., Song J., Chong Y., Wang J., Zhao G. (2019). miR-486-5p inhibits inflammatory response, matrix degradation and apoptosis of nucleus pulposus cells through directly targeting FOXO1 in intervertebral disc degeneration. Cell. Physiol. Biochem..

[130] Wang R., Wen B., Sun D. (2019). miR-573 regulates cell proliferation and apoptosis by targeting Bax in nucleus pulposus cells. Cell Mol. Biol. Lett..

[131] Zhan B., Zhan Y., Wang W., Zhan Y., Liu B. (2018). Expression of miR-625 and Fas in cervical vertebral cartilage endplate. Exp. Ther. Med..

[132] Tan H., Zhao L., Song R., Liu Y., Wang L. (2018). microRNA-665 promotes the proliferation and matrix degradation of nucleus pulposus through targeting GDF5 in intervertebral disc degeneration. J. Cell. Biochem..

[133] Chen Z., Han Y., Deng C., Chen W., Jin L., Chen H., Wang K., Shen H., Qian L. (2019). Inflammation-dependent downregulation of miR-194-5p contributes to human intervertebral disc degeneration by targeting CUL4A and CUL4B. J. Cell. Physiol..

[134] Liu W., Zhang Y., Xia P., Li S., Feng X., Gao Y., Wang K., Song Y., Duan Z., Yang S. (2016). MicroRNA-7 regulates IL-1β-induced extracellular matrix degeneration by targeting GDF5 in human nucleus pulposus cells. Biomed. Pharmacother..

[135] Wang B., Wang D., Yan T., Yuan H. (2016). MiR-138-5p promotes TNF-alpha-induced apoptosis in human intervertebral disc degeneration by targeting SIRT1 through PTEN/PI3K/Akt signaling. Exp. Cell Res..

[136] Stanczyk J., Pedrioli D.M., Brentano F., Sanchez-Pernaute O., Kolling C., Gay R.E., Detmar M., Gay S., Kyburz D. (2008). Altered expression of MicroRNA in synovial fibroblasts and synovial tissue in rheumatoid arthritis. Arthritis Rheum..

[137] Gruber H.E., Norton H.J., Ingram J.A., Hanley E.N. (2005). The SOX9 transcription factor in the human disc: Decreased immunolocalization with age and disc degeneration. Spine.

[138] Pauley K.M., Satoh M., Chan A.L., Bubb M.R., Reeves W.H., Chan E.K. (2008). Upregulated miR-146a expression in peripheral blood mononuclear cells from rheumatoid arthritis patients. Arthritis Res. Ther..

[139] Tomaselli S., Panera N., Gallo A., Alisi A. (2012). Circulating miRNA profiling to identify biomarkers of dysmetabolism. Biomark. Med..

[140] Trefilova V.V., Shnayder N.A., Novitsky M.A., Ovdienko O.A., Nurgaliev Z.A. (2022). Application of patient-reported outcomes in back pain in adults: Part 1. Pers. Psychiatry Neurol..

[141] Chen S., Wang Y., Wu H., Fang X., Wang C., Wang N., Xie L. (2023). Research hotspots and trends of microRNAs in intervertebral disc degeneration: A comprehensive bibliometric analysis. J. Orthop. Surg. Res..

[142] Yang F., Wang J., Chen Z., Yang Y., Zhang W., Guo S., Yang Q. (2021). Role of microRNAs in intervertebral disc degeneration (Review). Exp. Ther. Med..

[143] Guo C., Chen Y., Wang Y., Hao Y. (2022). Regulatory roles of noncoding RNAs in intervertebral disc degeneration as potential therapeutic targets (Review). Exp. Ther. Med..

[144] Guo Y., Tian L., Liu X., He Y., Chang S., Shen Y. (2019). ERRFI1 Inhibits Proliferation and Inflammation of Nucleus Pulposus and Is Negatively Regulated by miR-2355-5p in Intervertebral Disc Degeneration. Spine.

[145] Song Q., Zhang F., Wang K., Chen Z., Li Q., Liu Z., Shen H. (2021). MiR-874-3p plays a protective role in intervertebral disc degeneration by suppressing MMP2 and MMP3. Eur. J. Pharmacol..

[146] Li Z., Tang Y., Wang L., Wang K., Huang S., Chen Y. (2024). Tetrahedral framework nucleic acids-based delivery of microRNA-155 alleviates intervertebral disc degeneration through targeting Bcl-2/Bax apoptosis pathway. Cell Prolif..

[147] Li X., Hou Q., Yuan W., Zhan X., Yuan H. (2023). Inhibition of miR-96-5p alleviates intervertebral disc degeneration by regulating the peroxisome proliferator-activated receptor γ/nuclear factor-kappaB pathway. J. Orthop. Surg. Res..

[148] Shi J., Wang S., He Q., Liu K., Zhao W., Xie Q., Cheng L. (2021). TNF-α induces up-regulation of MicroRNA-27a via the P38 signalling pathway, which inhibits intervertebral disc degeneration by targeting FSTL1. J. Cell. Mol. Med..

[149] Hu B., Xu C., Tian Y., Shi C., Zhang Y., Deng L., Zhou H., Cao P., Chen H., Yuan W. (2017). Inflammatory microRNA-194 and -515 attenuate the biosynthesis of chondroitin sulfate during human intervertebral disc degeneration. Oncotarget.

[150] Bin S., Xin L., Lin Z., Jinhua Z., Rui G., Xiang Z. (2021). Targeting miR-10a-5p/IL-6R axis for reducing IL-6-induced cartilage cell ferroptosis. Exp. Mol. Pathol..

[151] Kong L., Sun M., Jiang Z., Li L., Lu B. (2018). MicroRNA-194 Inhibits Lipopolysaccharide-Induced Inflammatory Response in Nucleus Pulposus Cells of the Intervertebral Disc by Targeting TNF Receptor-Associated Factor 6 (TRAF6). Med. Sci. Monit..

[152] Zhang Q.C., Hu S.Q., Hu A.N., Zhang T.W., Jiang L.B., Li X.L. (2021). Autophagy-activated nucleus pulposus cells deliver exosomal miR-27a to prevent extracellular matrix degradation by targeting MMP-13. J. Orthop. Res..

[153] Lin X., Lin Q. (2020). MiRNA-495-3p Attenuates TNF-α Induced Apoptosis and Inflammation in Human Nucleus Pulposus Cells by Targeting IL5RA. Inflammation.

[154] Dong L., Dong B. (2021). miR-489-3p overexpression inhibits lipopolysaccharide-induced nucleus pulposus cell apoptosis, inflammation and extracellular matrix degradation via targeting Toll-like receptor 4. Exp. Ther. Med..

[155] Zhou J., Liang A., Hong J., Sun J., Lin X., Peng Y., Wang X., Sun S., Xiao D., Xu K. (2019). MicroRNA-155 suppresses the catabolic effect induced by TNF-α and IL-1β by targeting C/EBPβ in rat nucleus pulposus cells. Connect. Tissue Res..

[156] Dmitrenko D.V. (2025). Personalized Medicine in Neurology. Pers. Psychiatry Neurol..

[157] Boon R.A., Vickers K.C. (2013). Intercellular transport of microRNAs. Arterioscler. Thromb. Vasc. Biol..

