# Supplementary material for: Alteration of microRNA Expression Associated with Chronic Back Pain in Patients with Intervertebral Disc Degeneration: A Scoping Review
Source: Int J Mol Sci. 2026 Jan 23;27(3):1167. doi: 10.3390/ijms27031167 (PMC12897208; doi:10.3390/ijms27031167)
Supplement: Supplementary file 1 [file ijms-27-01167-s001.zip › ijms-4045174-supplementary.pdf]

Preferred Reporting Items for Systematic reviews and Meta-Analyses extension for Scoping Reviews (PRISMA-ScR) Checklist

SECTION	ITEM	PRISMA-ScR CHECKLIST ITEM	REPORTED ON PAGE #
**TITLE**			
Title	1	Identify the report as a scoping review.	Alteration of microRNA Expression Associated with Chronic Back Pain in Patients with Intervertebral Disc Degeneration: Scoping Review
**ABSTRACT**			
Structured summary	2	Provide a structured summary that includes (as applicable): background, objectives, eligibility criteria, sources of evidence, charting methods, results, and conclusions that relate to the review questions and objectives.	Background: Chronic back pain (CBP) associated with intervertebral disc degeneration (IVDD) is a leading cause of medical consultations, decreased quality of life, and temporary and permanent disability. The mechanisms of CBP development and persistence in patients with IVDD have been studied for many years, but this issue remains far from resolved. The search for predictive biomarkers that could help identify patients with IVDD at high risk for CBP continues. In recent decades, research has shown increasing interest in identifying epigenetic biomarkers for this disorder. Objective: to summarize the results of preclinical and clinical studies on the role of microRNAs as epigenetic biomarkers of the development and progression of CBP in patients with IVDD. Eligibility criteria: original experimental (preclinical) studies; original clinical study; assessment of changes in systemic (in blood) and/or local (in IVD) levels of microRNA expression in IDD, either independently or in comparison with healthy controls; studies that were completed and the results of which were published. Sources of evidence: PubMed, Springer, Google Scholar, Scopus, Oxford Press, Cochrane, and e-Library data-bases. Charting methods: Charting for this scoping review involved developing a data extraction form to summarize extract and organize data from included studies. This was an iterative process where the charting tables and figures may be refined as the review progresses. Results: 126 studies were analyzed in detail, focusing on their study designs and comparing changes in microRNA ex-pression in animal models of IVDD and in patients with IVDD compared to healthy controls.

SECTION	ITEM	PRISMA-ScR CHECKLIST ITEM	REPORTED ON PAGE #
			During the preparation of this scoping review and upon subsequent detailed review of the original publications, it turned out that the results of one study were not justified by the authors due to identified technological problems (the article was withdrawn by the editorial board of the journal). Therefore, we excluded the results of this study from the subsequent analysis. As a result, this section summarizes the results of 60 preclinical and 65 clinical studies. Some miRNAs (e.g., miR-21 and miR-132) are associated with the regulation of inflammatory pathways that contribute to increased degradation of IVD extracellular matrix and enhanced nociceptive signaling through various mechanisms, contributing to the progression of CBP. Other microRNAs (e.g., miR-145, miR-223) exert protective effects, enhance regenerative potential, and alleviate CBP. Despite the promising results of these studies, there are limitations in the use of microRNAs as promising epigenetic biomarkers of CBP in patients with IVDD because the pattern of predictive and protective microRNAs in relation to the mechanisms of CBP formation and progression in IVDD has not yet been sufficiently studied. Conclusions. The results of some preclinical and clinical studies are contradictory. Further research is needed to clarify the role of microRNAs in animal models in clinical trials. Further research may open new opportunities for targeted therapy based on miRNA-based epigenetic changes in CBP in patients with IVDD as one of the approaches to high-tech medical care.
**INTRODUCTION**			
Rationale	3	Describe the rationale for the review in the context of what is already known. Explain why the review questions/objectives lend themselves to a scoping review approach.	The rationale for this scoping review was the increasing evidence that microRNAs are involved in the pathological processes of IVDD (including pain and inflammation), making them promising candidates for epigenetic diagnostic biomarkers and therapeutic targets. While many miRNAs have been implicated in IVDD through their role in apoptosis, ECM remodeling, and inflammation, further testing is needed to identify specific miRNAs for early

SECTION	ITEM	PRISMA-ScR CHECKLIST ITEM	REPORTED ON PAGE #
			diagnosis, stratify patients with IVDD (high and medium risk groups), develop new treatments (disease-modulated miRNA as drugs of new generation), and confirm their causal role in developing CBP in patients with IVDD.
Objectives	4	Provide an explicit statement of the questions and objectives being addressed with reference to their key elements (e.g., population or participants, concepts, and context) or other relevant key elements used to conceptualize the review questions and/or objectives.	The aim of this scoping review is to summarize the results of preclinical and clinical studies on the role of microRNAs as epigenetic biomarkers of the development and progression of CBP in patients with IVDD.
**METHODS**			
Protocol and registration	5	Indicate whether a review protocol exists; state if and where it can be accessed (e.g., a Web address); and if available, provide registration information, including the registration number.	Verification of the results was carried out by the second corresponding author, who acted as an external expert (M.A.-Z.). There is no verification protocol, as it is not necessary but desirable for review reviews (unlike systematic reviews).
Eligibility criteria	6	Specify characteristics of the sources of evidence used as eligibility criteria (e.g., years considered, language, and publication status), and provide a rationale.	English-language articles; original experimental (preclinical) studies; original clinical study; assessment of changes in systemic (in blood) and/or local (in IVD) levels of microRNA expression in IDD, either independently or in comparison with healthy controls; studies that were completed and the results of which were published.
Information sources*	7	Describe all information sources in the search (e.g., databases with dates of coverage and contact with authors to identify additional sources), as well as the date the most recent search was executed.	A search was conducted in the PubMed, Springer, Google Scholar, Scopus, Oxford Press, Cochrane, and e-Library databases. Publications from 2015 to 2025 were analyzed, including original clinical studies involving patients with CBP and signs of systemic inflammatory response syndrome. In addition, publications of clinical and historical interest (published no earlier than 2005) were included.
Search	8	Present the full electronic search strategy for at least 1 database, including any limits used, such that it could be repeated.	The search was conducted using the following keywords and phrases: microRNA, intervertebral disc degeneration, chronic pain syndrome, low back pain, epigenetics, systemic inflammatory response syndrome, diagnosis and treatment.

SECTION	ITEM	PRISMA-ScR CHECKLIST ITEM	REPORTED ON PAGE #
Selection of sources of evidence†	9	State the process for selecting sources of evidence (i.e., screening and eligibility) included in the scoping review.	We excluded articles that were case reports or reviews, as well as studies that were still ongoing.
Data charting process‡	10	Describe the methods of charting data from the included sources of evidence (e.g., calibrated forms or forms that have been tested by the team before their use, and whether data charting was done independently or in duplicate) and any processes for obtaining and confirming data from investigators.	The methods of plotting data from the included evidence sources (including tables and figures) were tested by the team before using them, and the tables and figures were compiled in duplicate.
Data items	11	List and define all variables for which data were sought and any assumptions and simplifications made.	Before charting, we determined the specific information needed to answer the review's objective. This included study details, samples characteristics, intervention types, and reported outcomes. Charting tables for this scoping review involved creating a standardized data extraction form to summarize collect and organize information from included studies. Also, we decided on the appropriate level of detail for each data point. Some information, like author and year, were standard, while other fields required more in-depth extraction. We considered using data visualizations like concept figures to present the findings of this review in a clear and logical manner. The charting process was iterative and was facilitated by specialized software BioRender (https://www.biorender.com/). As we chart, we were identified additional useful data points that can be added to the figures, continually updating it as the review progresses.
Critical appraisal of individual sources of evidence§	12	If done, provide a rationale for conducting a critical appraisal of included sources of evidence; describe the methods used and how this information was used in any data synthesis (if appropriate).	This scoping review was based on the study and synthesis of available publications relevant to the topic and purpose of our study, identifying key concepts, theories, and gaps in microRNA research as epigenetic biomarkers of pain and inflammation in patients with intervertebral disc degeneration. Unlike other types of reviews (in particular, a systematic review or meta-analysis), our scoping review focused on reviewing and mapping the entire data set, rather than answering a specific research question,

SECTION	ITEM	PRISMA-ScR CHECKLIST ITEM	REPORTED ON PAGE #
			that is, it was used as the first step to determine the directions of future research.
Synthesis of results	13	Describe the methods of handling and summarizing the data that were charted.	We did not use statistical data processing and plotting methods, since the analyzed preclinical and clinical studies were characterized by great variability in the design, type and sample sizes.
**RESULTS**			
Selection of sources of evidence	14	Give numbers of sources of evidence screened, assessed for eligibility, and included in the review, with reasons for exclusions at each stage, ideally using a flow diagram.	The number of verified evidence sources assessed for compliance and included in the review, with reasons for exclusion at each stage, is presented in this systematic review using a flow chart (Figure 1).
Characteristics of sources of evidence	15	For each source of evidence, present characteristics for which data were charted and provide the citations.	For each source of factual data, we have indicated the characteristics for which the data was collected and provided links to them (Tables 1 and 2).
Critical appraisal within sources of evidence	16	If done, present data on critical appraisal of included sources of evidence (see item 12).	Based on the inclusion and exclusion criteria, we analyzed 126 studies. The studies were analyzed in detail, focusing on their study designs and comparing changes in microRNA expression in animal models of IVDD and in patients with IVDD compared to healthy controls. During the preparation of this scoping review and upon subsequent de-tailed review of the original publications, it turned out that the results of one study were not justified by the authors due to identified technological problems (the article was with-drawn by the editorial board of the journal). Therefore, we excluded the results of this study from the subsequent analysis. As a result, this section summarizes the results of 60 preclinical and 65 clinical studies.
Results of individual sources of evidence	17	For each included source of evidence, present the relevant data that were charted that relate to the review questions and objectives.	For each included source of evidence, relevant data was provided, which were plotted on tables 1 and 2, and which related to the issues and objectives of verification.
Synthesis of results	18	Summarize and/or present the charting results as they relate to the review questions and objectives.	The results have been summarized and presented in figures in the form in which they relate to the issues and objectives of our review.
**DISCUSSION**			
Summary of evidence	19	Summarize the main results (including an overview of concepts, themes, and types of evidence available), link to	The main results were summarized, references were given to the issues and objectives of the review, and their

SECTION	ITEM	PRISMA-ScR CHECKLIST ITEM	REPORTED ON PAGE #
		the review questions and objectives, and consider the relevance to key groups.	relevance to key groups of patients with IVDD was reviewed.
Limitations	20	Discuss the limitations of the scoping review process.	The limitations of this scoping review are discussed.
Conclusions	21	Provide a general interpretation of the results with respect to the review questions and objectives, as well as potential implications and/or next steps.	A general interpretation of the results is provided regarding the issues and purpose of this scoping review, as well as potential implications and/or next steps in the future.
**FUNDING**			
Funding	22	Describe sources of funding for the included sources of evidence, as well as sources of funding for the scoping review. Describe the role of the funders of the scoping review.	This scoping review did not have sources of funding for the evidence sources included, nor did it have a source of funding for the review.

JBI = Joanna Briggs Institute; PRISMA-ScR = Preferred Reporting Items for Systematic reviews and Meta-Analyses extension for Scoping Reviews.
* Where *sources of evidence* (see second footnote) are compiled from, such as bibliographic databases, social media platforms, and Web sites.
† A more inclusive/heterogeneous term used to account for the different types of evidence or data sources (e.g., quantitative and/or qualitative research, expert opinion, and policy documents) that may be eligible in a scoping review as opposed to only studies. This is not to be confused with *information sources* (see first footnote).
‡ The frameworks by Arksey and O'Malley (6) and Levac and colleagues (7) and the JBI guidance (4, 5) refer to the process of data extraction in a scoping review as data charting.
§ The process of systematically examining research evidence to assess its validity, results, and relevance before using it to inform a decision. This term is used for items 12 and 19 instead of "risk of bias" (which is more applicable to systematic reviews of interventions) to include and acknowledge the various sources of evidence that may be used in a scoping review (e.g., quantitative and/or qualitative research, expert opinion, and policy document).

From: Tricco AC, Lillie E, Zarin W, O'Brien KK, Colquhoun H, Levac D, et al. PRISMA Extension for Scoping Reviews (PRISMAScR): Checklist and Explanation. Ann Intern Med. 2018;169:467–473. doi: 10.7326/M18-0850.
